# Rationale and design of the PeriOperative ISchemic Evaluation-3 (POISE-3): a randomized controlled trial evaluating tranexamic acid and a strategy to minimize hypotension in noncardiac surgery

**DOI:** 10.1186/s13063-021-05992-1

**Published:** 2022-01-31

**Authors:** Maura Marcucci, Thomas W. Painter, David Conen, Kate Leslie, Vladimir V. Lomivorotov, Daniel Sessler, Matthew T. V. Chan, Flavia K. Borges, Maria J. Martínez Zapata, C. Y. Wang, Denis Xavier, Sandra N. Ofori, Giovanni Landoni, Sergey Efremov, Ydo V. Kleinlugtenbelt, Wojciech Szczeklik, Denis Schmartz, Amit X. Garg, Timothy G. Short, Maria Wittmann, Christian S. Meyhoff, Mohammed Amir, David Torres, Ameen Patel, Emmanuelle Duceppe, Kurtz Ruetzler, Joel L. Parlow, Vikas Tandon, Michael K. Wang, Edith Fleischmann, Carisi A. Polanczyk, Raja Jayaram, Sergey V. Astrakov, Mangala Rao, Tomas VanHelder, William K. K. Wu, Chao Chia Cheong, Sabry Ayad, Marat Abubakirov, Mikhail Kirov, Keyur Bhatt, Miriam de Nadal, Valery Likhvantsev, Pilar Paniagua Iglesisas, Hector J. Aguado, Michael McGillion, Andre Lamy, Richard P. Whitlock, Pavel Roshanov, David Stillo, Ingrid Copland, Jessica Vincent, Kumar Balasubramanian, Shrikant I. Bangdiwala, Bruce Biccard, Andrea Kurz, Sadeesh Srinathan, Shirley Petit, John Eikelboom, Toby Richards, Peter L. Gross, Pascal Alfonsi, Gordon Guyatt, Emily Belley-Cote, Jessica Spence, William McIntyre, Salim Yusuf, P. J. Devereaux

**Affiliations:** 1 Department of Health Research Methods, Evidence, and Impact, McMaster University, 237 Barton Street East, Hamilton, ON L8L 2X2 Canada; 2 Department of Medicine, McMaster University, 237 Barton Street East, Hamilton, ON L8L 2X2 Canada; 3 Population Health Research Institute, Hamilton, ON Canada; 4 Discipline of Acute Care Medicine, University of Adelaide, Adelaide, South Australia Australia; 5 Department of Anaesthesia, Royal Adelaide Hospital, Adelaide, South Australia Australia; 6 Department of Anaesthesia and Pain Management, Royal Melbourne Hospital, Melbourne, Australia; 7 Department of Critical Care Medicine, Melbourne Medical School, University of Melbourne, Melbourne, Australia; 8 Department of Anesthesiology and Intensive Care, E. Meshalkin National Medical Research Center, Novosibirsk, Russia; 9 Department of Anesthesiology and Intensive Care, Novosibirsk State University, Novosibirsk, Russia; 10 Department of Outcomes Research, Anesthesiology Institute, Cleveland Clinic, Cleveland, Ohio USA; 11 The Chinese University of Hong Kong, Hong Kong, China; 12 Iberoamerican Cochrane Centre-Public Health and Clinical Epidemiology Service, IIB Sant Pau, CIBER de Epidemiología y Salud Pública (CIBERESP), Barcelona, Spain; 13 Department of Anaesthesiology, Faculty of Medicine, University of Malaya, Kuala Lumpur, Malaysia; 14 St. John’s Medical College, Bangalore, India; 15 Department of Anesthesia and Intensive Care, IRCCS San Raffaele Scientific Institute, Milan, Italy; 16 School of Medicine, Vita-Salute San Raffaele University, Milan, Italy; 17 Saint Petersburg State University Hospital, Saint Petersburg, Russia; 18 Department of Orthopedic and Trauma Surgery, Deventer Ziekenhuis, Deventer, the Netherlands; 19 Jagiellonian University Medical College, Center for Intensive Care and Perioperative Medicine, Krakow, Poland; 20 CHU Brugmann, Université libre de Bruxelles, Brussels, Belgium; 21 Department of Medicine, Epidemiology and Biostatistics, Western University, London, ON Canada; 22 Auckland City Hospital, Auckland, New Zealand; 23 University of Auckland, School of Health Sciences, Auckland, New Zealand; 24 Department of Anesthesiology, University Hospital, Bonn, Germany; 25 Department of Anaesthesia and Intensive Care, Bispebjerg and Frederiksberg Hospital, University of Copenhagen, Copenhagen, Denmark; 26 Shifa International Hospital (STMU), Islamabad, Pakistan; 27 Clinica Santa Maria, Santiago, Chile; 28 Universidad de Los Andes, Santiago, Chile; 29 Department of Medicine, Universite de Montreal, Montreal, QC Canada; 30 Department of Anesthesiology and Perioperative Medicine, Kingston General Hospital and Queen’s University, Kingston, ON Canada; 31 Department of Anesthesia, General Intensive Care and Pain Management, Medical University of Vienna, Vienna, Austria; 32 Universidade Federal do Rio Grande do Sul, Porto Alegre, Brazil; 33 Cardiology Department, Hospital de Clínicas de Porto Alegre, Porto Alegre, Brazil; 34 Nuffield Department of Anaesthetics, Clinical Neurosciences, University of Oxford, Oxford, UK; 35 City hospital No. 25, Novosibirsk, Russia; 36 Department of Anesthesiology, Novosibirsk State University, Novosibirsk, Russia; 37 Department of Anesthesia, McMaster University, Hamilton, ON Canada; 38 Case Western Reserve University, Anesthesiology Institute, Cleveland Clinic - Fairview Hospital, Cleveland, OH USA; 39 Meshalkin National Medical Research Center, Novosibirsk, Russia; 40 Department of Anesthesiology and Intensive Care Medicine, Northern State Medical University, Arkhangelsk, Russia; 41 SIDS Hospital & Research Centre, Surat, India; 42 Anesthesiology and Intensive Care Department, Hospital Universitari Vall d’Hebron, Barcelona, Spain; 43 V. Negovskiy Reanimatology Research Institute, Moscow, Russia; 44 M. Vladimirskiy Moscow Regional Clinical and Research Institute, Moscow, Russia; 45 Department of Anaesthesia and Pain Management Santa Creu i Sant Pau University Hospital, Barcelona, Spain; 46 Trauma & Orthopaedic surgery department, Hospital Clínico Universitario, Valladolid, Spain; 47 School of Nursing, McMaster University, Hamilton, ON Canada; 48 Department of Surgery, McMaster University, Hamilton, ON Canada; 49 Department of Medicine, London Health Sciences Centre, London, ON Canada; 50 Department of Anaesthesia and Perioperative Medicine, Groote Schuur Hospital and University of Cape Town, Cape Town, South Africa; 51 Department of General Anaesthesiology, Emergency- and Intensive Care Medicine, Medical University Graz, Graz, Austria; 52 Department of Surgery, University of Manitoba, Winnipeg, Manitoba Canada; 53 Faculty of Health and Medical Sciences, University of Western Australia, Perth, Australia; 54 Department of Anesthesiology, GH Paris Saint Joseph, Paris, France

**Keywords:** Noncardiac surgery, Tranexamic acid, Perioperative bleeding, Perioperative hypotension, Cardiovascular complications, Randomized controlled trial

## Abstract

**Background:**

For patients undergoing noncardiac surgery, bleeding and hypotension are frequent and associated with increased mortality and cardiovascular complications. Tranexamic acid (TXA) is an antifibrinolytic agent with the potential to reduce surgical bleeding; however, there is uncertainty about its efficacy and safety in noncardiac surgery. Although usual perioperative care is commonly consistent with a hypertension-avoidance strategy (i.e., most patients continue their antihypertensive medications throughout the perioperative period and intraoperative mean arterial pressures of 60 mmHg are commonly accepted), a hypotension-avoidance strategy may improve perioperative outcomes.

**Methods:**

The PeriOperative Ischemic Evaluation (POISE)-3 Trial is a large international randomized controlled trial designed to determine if TXA is superior to placebo for the composite outcome of life-threatening, major, and critical organ bleeding, and non-inferior to placebo for the occurrence of major arterial and venous thrombotic events, at 30 days after randomization. Using a partial factorial design, POISE-3 will additionally determine the effect of a hypotension-avoidance strategy versus a hypertension-avoidance strategy on the risk of major cardiovascular events, at 30 days after randomization. The target sample size is 10,000 participants. Patients ≥45 years of age undergoing noncardiac surgery, with or at risk of cardiovascular and bleeding complications, are randomized to receive a TXA 1 g intravenous bolus or matching placebo at the start and at the end of surgery. Patients, health care providers, data collectors, outcome adjudicators, and investigators are blinded to the treatment allocation. Patients on ≥ 1 chronic antihypertensive medication are also randomized to either of the two blood pressure management strategies, which differ in the management of patient antihypertensive medications on the morning of surgery and on the first 2 days after surgery, and in the target mean arterial pressure during surgery. Outcome adjudicators are blinded to the blood pressure treatment allocation. Patients are followed up at 30 days and 1 year after randomization.

**Discussion:**

Bleeding and hypotension in noncardiac surgery are common and have a substantial impact on patient prognosis. The POISE-3 trial will evaluate two interventions to determine their impact on bleeding, cardiovascular complications, and mortality.

**Trial registration:**

ClinicalTrials.gov NCT03505723. Registered on 23 April 2018.

**Supplementary Information:**

The online version contains supplementary material available at 10.1186/s13063-021-05992-1.

## Introduction

Perioperative bleeding in noncardiac surgery is frequent and is associated with a poor prognosis. In the Vascular events In noncardiac Surgery patIents cOhort evaluatioN (VISION) study—a large, international, prospective cohort study that included a representative sample of 40,000 adults ≥ 45 years of age undergoing noncardiac surger y[1, 2]—major bleeding was the complication with the highest attributable risk for mortality at 30 days, accounting for 17% of the deaths [3]. Perioperative bleeding is also independently associated with cardiovascular complications including myocardial injury and infarction, stroke, and acute kidney injury [4–7].

Tranexamic acid (TXA) is an antifibrinolytic agent that has been shown to safely prevent clinically important bleeding in large randomized controlled trials (RCTs) in various settings, including acute trauma [8], obstetrics [9], and cardiac surgery [10]. However, a recent large RCT that evaluated TXA in patients with acute gastrointestinal bleeding reported an increased risk of venous thromboembolism with TXA versus placebo (48 [0.8%] of 5952 patients versus 26 [0.4%] of 5977 patients; relative risk [RR], 1.85; 95% confidence interval [CI], 1.15–2.98) [11]. The risk-benefit profile of TXA in those undergoing noncardiac surgery is unknown. Several small RCTs in orthopedic surgery have suggested that TXA reduces blood loss and transfusions [12–16]. A few small RCTs have been conducted in other types of noncardiac surgery [17, 18]. Although there is concern that TXA may increase thrombotic events in a prothrombotic setting such as noncardiac surgery, if TXA prevents bleeding, it may reduce cardiovascular complications. The existing studies of TXA in noncardiac surgery were too small—and often selected a low-risk population—to definitely establish its cardiovascular safety [19]. We conducted a pilot study in 100 patients, with or at risk of cardiovascular disease, undergoing noncardiac surgery at two sites in Hamilton, ON, Canada [20]. Patients were randomized to receive TXA or matching placebo, as an intravenous bolus of 1 g at the beginning and at the end of surgery. We demonstrated the feasibility of recruiting such a patient population and of administering the study drug [20].

Perioperative hypertension has been associated with cardiovascular complications after noncardiac surgery [21–24]. Perioperative hypotension occurs most frequently in the operating room, but it is also common during the first two days after surgery; moreover,  on surgical wards, hypotension lasts longer than in the intensely monitored operating room [25, 26]. Preoperative, intraoperative, and postoperative hypotension are independently associated with an increased risk of all-cause mortality and cardiovascular complications at 30 days after noncardiac surgery [25–31]. Two RCTs suggested benefits of higher or individualized perioperative blood pressure (BP) targets [32, 33]; however, these trials were relatively small with few events [32, 33].

Usual perioperative care is commonly consistent with a hypertension-avoidance strategy, that is most patients continue their antihypertensive medications throughout the perioperative period and low intraoperative mean arterial pressures (MAPs) are commonly accepted [25]. Observational studies and small RCTs suggest that withholding antihypertensive medications, and in particular angiotensin converting enzyme inhibitors (ACEIs) or angiotensin receptor blockers (ARBs), may reduce perioperative hypotension and cardiovascular complications [25, 34–36]. There is, however, no definitive evidence from adequately powered RCTs to inform whether a hypotension-avoidance or hypertension-avoidance strategy is superior.

We designed the third PeriOperative ISchemic Evaluation (POISE-3) Trial to address the following questions in patients with or at risk of cardiovascular disease who are undergoing noncardiac surgery: (1) is TXA superior to placebo for the occurrence of life-threatening, major, and critical organ bleeding, and non-inferior to placebo for the occurrence of major arterial and venous thrombotic events, within 30 days after surgery? and (2) among patients also chronically taking an antihypertensive drug, is a perioperative hypotension-avoidance strategy superior to a hypertension-avoidance strategy on the 30-day risk of a major cardiovascular event?

## Methods

### Trial design

POISE-3 is an international RCT of 10,000 adults at risk of bleeding and cardiovascular complications who are undergoing noncardiac surgery. Patients are randomized to receive intraoperative TXA or placebo. Using a 2 × 2 partial factorial design, patients taking ≥ 1 antihypertensive medication are also randomized to a hypotension-avoidance or a hypertension-avoidance strategy. The trial is registered at clinicaltrials.gov (NCT03505723). The Standard Protocol Items: Recommendations for

Interventional Trials (SPIRIT) checklist for our paper is provided as Additional file 1.

### Trial population

We include patients who meet the following criteria: (1) undergoing noncardiac surgery; (2) ≥ 45 years of age; (3) expected to stay in hospital at least one night after surgery; (4) meeting ≥ 1 of 6 cardiovascular and bleeding risk criteria (Table 1); and (5) providing written informed consent. Detailed inclusion criteria including definitions are provided in the Additional File 2. Table 2 describes the exclusion criteria.

**Table 1:** Inclusion criteria

Patients must fulfill ≥ 1 of the following criteria:	
1. NT-proBNP ≥ 200 ng/L;	
2. History of coronary artery disease;	
3. History of peripheral arterial disease;	
4. History of stroke;	
5. Undergoing major vascular surgery; OR	
6. Any 3 of 9 risk criteria A. Undergoing major surgery; B. History of congestive heart failure; C. History of a transient ischemic attack; D. Diabetes and currently taking an oral hypoglycemic agent or insulin; E. Age ≥ 70 years; F. History of hypertension; G. Serum creatinine > 175 μmol/L (> 2.0 mg/dl); H. History of smoking within 2 years of surgery; or I. Undergoing emergent/urgent surgery.	

*NT-proBNP* N-terminal pro–B-type natriuretic peptide

**Table 2:** Exclusion criteria

A. Patients are not eligible for POISE-3 trial if any of the following criteria is present:	
1. Patients undergoing cardiac surgery 2. Patients undergoing cranial neurosurgery 3. Planned use of systemic TXA during surgery 4. Low-risk surgical procedure (based on individual physician’s judgment) 5. Hypersensitivity or known allergy to TXA 6. Creatinine clearance < 30 mL/min (Cockcroft-Gault equation) or on chronic dialysis 7. History of seizure disorder 8. Patients with recent (< 3 months) stroke, myocardial infarction, acute arterial thrombosis, or venous thromboembolism 9. Patients with fibrinolytic condition following consumption coagulopathy 10. Patients with subarachnoid hemorrhage within the past 30 days 11. Women of childbearing potential who are not taking effective contraception, pregnant or breast-feeding 12. Previously enrolled in POISE-3 Trial	
B. Patients are not eligible for the BP management factorial trial if any of the following criteria is present:	
1. Patients with advanced congestive heart failure (New York Heart Association functional class III or IV or left ventricular ejection fraction ≤ 30%) 2. Patients with untreated brain aneurysm 3. Patients with previous history of hypertensive related cerebral hemorrhage 4. Patients undergoing surgery for pheochromocytoma or history of untreated pheochromocytoma 5. Patients who are hemodynamically unstable or requiring vasopressors or inotropic support before undergoing surgery 6. Patients with thyrotoxicosis (i.e., severe hyperthyroidism) requiring perioperative beta-blocker therapy	

*POISE* PeriOperative ISchemic Evaluation, *TXA* tranexamic acid, *BP* blood pressure

Patients are eligible for the BP management factorial if they are treated chronically (i.e., at least 30 days in the 6 weeks preceding randomization) with at least one antihypertensive medication of any class. Table 2 lists the additional exclusion criteria for the BP management factorial.

### Patient recruitment

In the majority of centers, study personnel screen the patient list in the preoperative assessment clinic to identify eligible patients. Multiple strategies are then applied in order to capture additional patients who do not attend the preoperative assessment clinic, including screening patients on the daily surgical list, patients on surgical wards and intensive care units, and patients in the preoperative holding area. At each center, the services of anesthesia, surgery, and medicine are requested to contact the study personnel regarding all surgical admissions through the emergency department and ward patients requiring surgery. Study personnel approach all eligible patients to obtain informed consent before surgery.

### Randomization and blinding

**Fig. 1:**
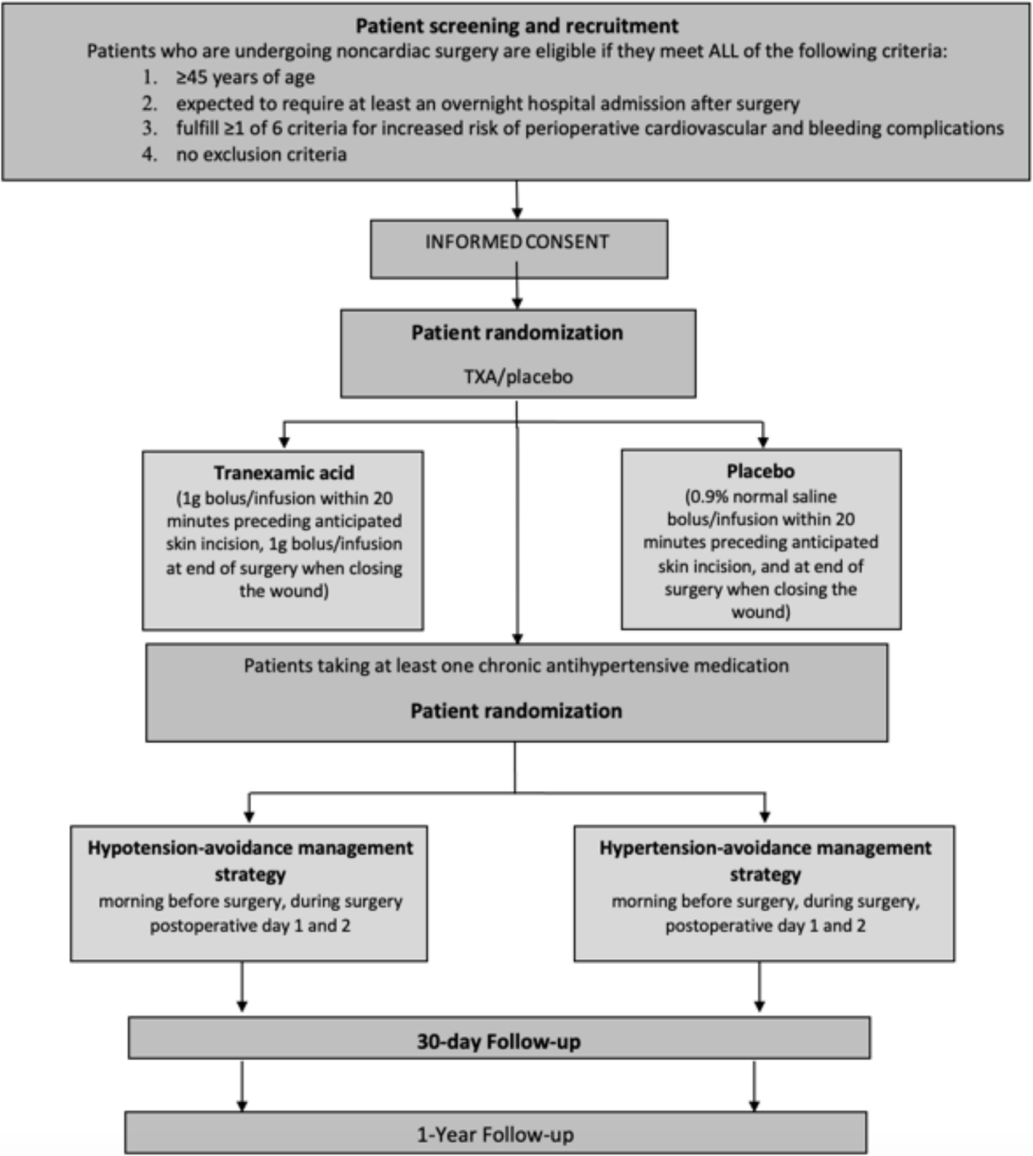
The POISE-3 trial flow chart

Figure 1 shows the trial flow chart. Study personnel randomize patients before surgery via a central 24-h Interactive Web Randomization System. The randomization process uses block randomization stratified by center, with block size varying randomly, and the study personnel do not know the block sizes. Patients are randomized to receive TXA or placebo according to a 1:1 ratio. Patients eligible for the BP management factorial are also randomized to the hypotension-avoidance or hypertension-avoidance strategy, with a 1:1 ratio.

Patients, health care providers, data collectors, outcome assessors and adjudicators, and investigators are all blinded to the TXA or placebo allocation. Outcome assessors and adjudicators are blinded to the treatment allocation for the comparison of BP management strategies.

We do not anticipate any requirement for unblinding of TXA treatment allocation; however, telephonic access (primary number and secondary back-up number) to a 24/7 central emergency unblinding system is provided to allow the blind to be broken when deemed absolutely necessary. Any unblinding will be recorded.

### Trial interventions

#### Tranexamic acid or placebo

Patients receive two intravenous doses of either 1 g of TXA or matching placebo (i.e., equivalent volume of 0.9% normal saline), as a bolus or 10-min infusion. The first dose is given at the beginning of surgery (i.e., within 20 min preceding the anticipated skin incision), and the second at the end of surgery (i.e., at wound closure). Additional File 3 provides the rationale for the choice of the TXA dosing regimen in this study.

TXA and 0.9% normal saline are sourced locally. Centers use any approved marketed version of TXA 100 mg/mL solution. The study drugs are prepared either by a pharmacist or another designated qualified personnel, who are not involved in any other study activities. In order to ensure blinding, individuals preparing study drugs at each participating center sign an agreement indicating they will maintain the confidentiality of randomization assignments.

#### Perioperative BP management strategies

9pt?>The BP strategies occur during three periods (i.e., preoperative, intraoperative, and postoperative), with the postoperative phase involving the first 2 days after the day of surgery. Whenever possible, patients identified as eligible for the BP management partial factorial are advised in advance not to take their antihypertensive medications on the morning of surgery or the night before surgery and to bring their antihypertensive medications to the hospital. In the preoperative and postoperative phase, patients’ vital signs are measured as per routine practice. Intraoperatively, sites are advised to monitor the MAP at least every 15 min.

##### Hypotension-avoidance BP management strategy (intervention)

**Fig. 2:**
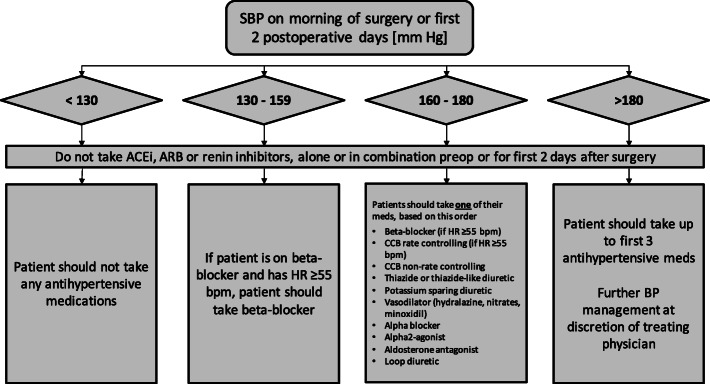
Algorithm for management of the patient antihypertensive medications in the hypotension avoidance strategy arm. SBP, systolic blood pressure; ACEi, angiotensin-converting-enzyme inhibitors; ARB, angiotensin II receptor blockers; HR, heart rate; CCB, calcium channel blockers; BP, blood pressure

In the hypotension-avoidance group, before the operation on the day of surgery, and during the first 2 days after surgery, the patient’s chronic antihypertensive therapy is managed based on a study algorithm, described in Fig. 2. Patients in this group are not given any ACEI, ARB, or renin inhibitor. Intraoperatively, the anesthesiologists are encouraged to target a MAP of ≥ 80 mmHg from the time of anesthetic induction until the end of surgery. Methods to achieve the intraoperative MAP target (e.g., fluids, vasopressors, inotropes) are left to the discretion of the attending anesthesiologist.

##### Hypertension-avoidance BP management strategy (control)

In the hypertension-avoidance group, on the morning of surgery before the operation, patients receive all the antihypertensive medications that they take chronically. The attending anesthesiologists are encouraged to target a MAP ≥ 60 mmHg from the time of anesthetic induction until the end of surgery. Patients resume taking their antihypertensive medications immediately after surgery. A POISE-3 iOS App has been developed and made available to participating sites to support the implementation of the BP management factorial (Additional File 4).

The Project Office reviews data on each center’s adherence to the interventions and provides feedback to centers to maximize center adherence. There are no special criteria for discontinuing or modifying allocated interventions for a given trial participant.

### Trial outcomes

#### TXA trial

The primary efficacy outcome is a composite of life-threatening, major, and critical organ bleeding at 30 days after randomization. The primary safety outcome is a composite of myocardial injury after noncardiac surgery (MINS) [2], non-hemorrhagic stroke, peripheral arterial thrombosis, and symptomatic proximal venous thromboembolism at 30 days after randomization.

The secondary outcomes at 30 days after randomization are as follows: (1) bleeding independently associated with mortality after noncardiac surgery (BIMS) [37]; (2) life-threatening bleeding; (3) major bleeding; (4) critical organ bleeding; (5) MINS; (63) MINS not fulfilling the universal definition of myocardial infarction [38]; (7) myocardial infarction; and (8) the composite of vascular death, bleeding (i.e., non-fatal life-threatening, major, or critical organ), MINS, stroke, peripheral arterial thrombosis, and symptomatic proximal venous thromboembolism (i.e. a net risk-benefit outcome).

#### BP management trial

The primary outcome is a composite of vascular death and non-fatal MINS, stroke, and cardiac arrest at 30 days after randomization.

The secondary outcomes at 30 days after randomization are as follows: (1) MINS; (2) MINS not fulfilling the universal definition of myocardial infarction [38]; (3) myocardial infarction; (4) stroke; (5) vascular mortality; and (6) all-cause mortality.

Tertiary outcomes at 30 days and 1 year are listed in the Additional File 5. All outcome definitions are listed in the Additional File 6. The Event Adjudication Committee consists of clinicians with expertise in perioperative outcomes who are blinded to treatment allocation and who will oversee the adjudication of the following outcomes: death (vascular versus non-vascular), MINS, myocardial infarction, cardiac arrest, stroke, peripheral arterial thrombosis, symptomatic pulmonary embolism, symptomatic proximal deep vein thrombosis, bleeding, acute congestive heart failure, new clinically important atrial fibrillation, acute kidney injury, infection/sepsis, and seizure.

### Follow-up

**Fig. 3:**
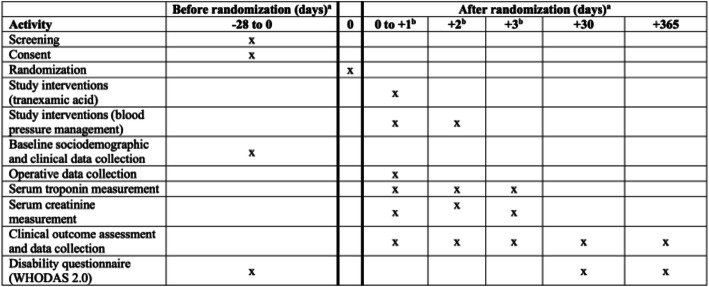
SPIRIT figure: participant timeline. Superscript lowercase letter “a” indicates the following: in most centers randomization occurs on the day of surgery prior to the procedure, and always within 24 h before the planned surgery. Superscript lowercase letter “b” indicates the following: in the table, days + 1, + 2, and + 3 refer to days + 1, + 2, and + 3 with respect to the day of surgery. Since randomization most often occurs on the day of surgery prior to the procedure, if surgery is not delayed or canceled, days + 1, + 2, and + 3 after randomization do correspond to postoperative days 1–3

The participant timeline based on the SPIRIT diagram is provided as Fig. 3. Study personnel follow patients throughout their time in hospital evaluating the patients and reviewing their medical records and recording any outcomes. For patients enrolled in the BP management factorial, study personnel ensure patients receive their antihypertensive medication as per the trial arm they were randomized to, up to postoperative day 2 inclusively. Study personnel contact all patients by telephone at 30 days and at 1 year after randomization to assess the occurrence of clinically relevant events that might meet any study outcome or serious adverse event definitions and to administer the disability questionnaire. In case the administration of the study interventions deviates from the protocol, study personnel continue to collect data on study outcomes at 30 days and at 1 year after randomization, unless the participants explicitly state that they do not want to be followed. If this happens, study personnel request to collect data on the patient through their healthcare provider. If the patient refuses this, no further data is collected on the patient.

### Data management

Study personnel at the participating sites record data on case report forms (CRFs) and submit the CRFs through a secure web-based computerized database (i.e., iDataFax). Patients are identified using a unique numeric code and all patient data are anonymized to ensure patient confidentiality. Data validity checks are programmed in the database and are monitored by data management assistants from the Project Office through multi-level data validation of CRFs.

### Statistical considerations

#### Sample size

The hypothesis of non-inferiority of TXA on thrombotic events compared with placebo is the one requiring the largest sample size. Table 3 shows the related sample size calculations. We expect the placebo event rate for the composite of arterial and venous thrombotic events to range between 10 and 12% (after accounting for the partial factorial design). We set up the non-inferiority margin on a hazard ratio (HR) with TXA compared with placebo of 1.125, which, given the expected placebo event rates, correspond to an absolute increase between 1.18 and 1.39%. The relative 12.5% hazard increase as non-inferiority margin was chosen to be half the 25% relative hazard reduction used to establish the superiority of either aspirin or clonidine compared with placebo in POISE-2 [5, 26]. Although we designed POISE-3 to establish if TXA is non-inferior to placebo, it is possible that TXA might in fact have a small beneficial effect on postoperative cardiovascular events due to the decreased bleeding. We quantified this small beneficial effect to correspond to a true HR of 0.9 (Table 3). Based on these assumptions, a sample size of 10,000 patients will allow us to test our non-inferiority hypothesis, with a power of ≥ 90% (Table 3).

**Table 3:** Sample size calculation for non-inferiority safety hypothesis of tranexamic acid vs. placebo

Placebo event rate	NI margin, absolute risk difference TXA vs. placebo^**b**^	Power	Sample size^**c**^
		0.90	8,868
**10%**^**a**^	1.18%	0.95	10,963
		0.97	12,441
		0.90	8,078
**11%**^**a**^	1.29%	0.95	9,990
		0.97	11,340
		0.90	7,390
**12%**^**a**^	1.39%	0.95	9,136
		0.97	10,367

*NI* non-inferiority margin, *TXA* tranexamic acid

^a^The estimates are based on the event rates observed in the POISE-2 trial (aspirin-placebo group) [5] and in a simulated subpopulation of the VISION cohort [1, 2] matching the POISE-3 inclusion criteria

^b^These are the NI margins expressed as absolute risk differences, corresponding to a NI margin expressed as hazard ratio of 1.125, when the placebo event rate is 10%, 11%, or 12%

^c^These sample sizes are obtained from non-inferiority test for two survival curves using Cox’s proportional hazard model, with a hazard ratio of 1.125 as non-inferiority margin, and a hazard ratio of 0.9 as actual hazard ratio (one-sided test, alpha of 0.025)

Taking into account information from the POISE-2 trial [5], we expect a placebo event rate for the TXA primary efficacy outcome of at least 7%. With a similar placebo event rate, a sample size of 10,000 patients will give us ≥ 95% power to show a relative hazard reduction of at least 30% (i.e., HR 0.70) with TXA compared to placebo in the composite of life-threatening, major and critical organ bleeding (based on Cox’s proportional hazard model, with type I error of 0.05, two-sided test). The trial will still have 90% power to detect a relative hazard reduction of at least 25% (i.e., HR 0.75).

We expect that at least 80% of patients will be on chronic antihypertensive therapy. We conservatively estimated that at least 65% of the patients in the TXA component of the trial would be eligible for the BP management factorial. A sample of 6500 patients will provide 95% power to test the hypothesis that the hypotension-avoidance strategy will reduce the occurrence of the composite of vascular death and nonfatal MINS, stroke, and cardiac arrest with an HR of 0.75 and an expected control event rate of 11.0% (based on Cox’s proportional hazard model, type I error of 0.05, two-sided test). The same sample size will still provide > 80% power to detect a HR of 0.80.

### Data analysis

For the TXA primary efficacy outcome, we will analyze patients in the treatment group to which they are allocated, according to the intention-to-treat principle. We will conservatively test the non-inferiority safety hypothesis in the per-protocol population [39]; a sensitivity intention-to-treat analysis will be secondarily performed. The analyses of the primary outcome in the BP management factorial will follow the intention-to-treat principle. Patients lost to follow-up will be censored at the time of their last follow-up. We will develop and finalize a statistical analysis plan before any investigator is unblinded.

#### Main analysis

For the analyses on each primary outcome, we will use Cox proportional hazards models with stratification according to the randomization in the partial factorial. We will assess model assumptions including the proportional hazard assumption. We will present the time-to-the first occurrence of one of the components of the primary outcomes using the Kaplan-Meier estimator.

For the effect of TXA on the primary safety outcome, we will calculate the HR and the corresponding upper bound of the one-sided 97.5% CI. We will declare the non-inferiority of TXA compared with placebo if the upper bound falls below 1.125.

For the effect of TXA compared with placebo on the primary efficacy outcome, and the effect of the hypotension-avoidance strategy compared with the hypertension-avoidance strategy on the primary outcome, we will calculate the HRs, corresponding 95% CIs and associated p-values. We will infer statistical significance if the computed 2-sided *p*-value is < 0.05. A similar approach will be adopted for the secondary and tertiary outcomes.

We anticipate that the effect of TXA and of the hypotension-avoidance strategy will act independently, but we will evaluate the possibility of synergism or antagonism between the two interventions by formally testing for differences among the strata.

#### Subgroup analyses

For the primary efficacy and safety outcomes in the TXA factorial, we will evaluate the following subgroups: orthopedic versus non-orthopedic surgery; preoperative hemoglobin < 120 g/L versus ≥ 120 g/L; preoperative estimated glomerular filtration rate (eGFR) < 45, 45– < 60, and ≥ 60 ml min^−1^ 1.73 m^2^; and preoperative N-terminal pro–B-type natriuretic peptide (NT-proBNP) < 200, 200– < 1500, and ≥ 1500 ng/L. We expect TXA to have greater benefit and safety in patients having orthopedic surgery, with a preoperative hemoglobin < 120 g/L, a lower preoperative eGFR, and higher preoperative NT-proBNP values.

For the primary outcome in the BP management factorial, we will evaluate the following subgroups: chronic ACEI/ARB therapy versus no chronic ACEI/ARB therapy; number of chronic antihypertensive medications (1 versus ≥ 2); preoperative systolic blood pressure (SBP) < 130, 130–159, 160–180, and > 180 mmHg; and preoperative NT-proBNP < 200, 200– < 1500, and ≥ 1500 ng/L. We expect the hypotension-avoidance strategy will have a greater beneficial effect in patients on chronic ACEI/ARB therapy, taking ≥ 2 chronic antihypertensive medications, with a lower preoperative SBP, and higher preoperative NT-proBNP values.

For each subgroup analysis, we will undertake a Cox proportional hazards model assessing each primary outcome incorporating a subgroup interaction term to provide the basis for evaluating subgroup effects. We will consider the possibility that a subgroup effect is present if the interaction term of treatment and subgroup is statistically significant at a *p*-value < 0.05. We will also consider other credibility criteria to judge the reliability of a subgroup effect [40, 41].

#### Interim analyses

POISE-3 interim analyses are described in the Additional File 7.

## Oversight and monitoring

### Trial organization

The Population Health Research Institute (PHRI; Hamilton General Hospital Campus, David Braley Cardiac, Vascular and Stroke Research Institute, 237 Barton Street East, Hamilton, Ontario, Canada L8L 2X2) is the sponsor and coordinating center for this trial and is responsible for the central randomization, trial database, data consistency checks, data analyses, and coordination of participating centers worldwide. Additional File 8 describes the trial organizational structure and the plan for the oversight of the trial conduct. Additional File 10 lists POISE-3 investigators across centres and the composition of the study committees.

### Adverse event reporting

In POISE-3 trial, we collect data on serious adverse events (SAEs) and suspected unexpected serious adverse reactions (SUSARs); however, we do not collect data on adverse events that are not serious. We defined an SAE as any untoward medical occurrence that at any dose is life-threatening, or requires inpatient hospitalization or prolongation of existing hospitalization, or results in persistent or significant disability/incapacity, or is a congenital anomaly/birth defect, or is a medically important event. We defined SUSARs as events that meet the following criteria: (1) suspected to be causally associated with TXA; (2) unexpected if the nature, severity, or outcome of the reaction(s) is not consistent with the reference information (i.e., product monograph for TXA); (3) serious (as defined above for an SAE); and (4) not a trial efficacy outcome.

Efficacy and safety outcomes will be recorded separately and not as SAEs, except if, because of the course or severity or any other feature of such events, the investigator, according to his/her best medical judgment, considers these events as exceptional in this medical condition. Hospitalizations, which were planned before inclusion in the study (e.g., elective or scheduled surgery or other interventions), will not be regarded as SAEs. This pertains also to hospitalizations which are part of the normal treatment or monitoring of the studied disease or another disease present before inclusion in the study (e.g., patient returning to the hospital for chemotherapy) and which did not result in a worsening of the disease.

All SAEs need to be reported within 24 h of knowledge of the event to the Project Office. For such events, research personnel will complete an SAE CRF in the database. The Project Office will then inform regulatory authorities in a timely manner, as necessary, according to the applicable regulations.

The Data Monitoring Committee (DMC) will provide oversight of patients’ safety throughout the trial by reviewing unblinded aggregate data (including all reported study outcome events and SAEs) by treatment group at regular intervals throughout the duration of the trial and as defined in the DMC Charter.

## Dissemination

Our dissemination plan includes the following: presentation at national and international conferences, publications in peer reviewed high-impact journals, and posts on the “Reducing Global Perioperative Risk” Resource Centre (http://perioperative-risk.amjmed.com/), a multimedia global-scale platform we developed with Elsevier (Canadian Institutes of Health Research funded), linked to Elsevier’s global online readership.

## Discussion

Over 200 million adults annually undergo major noncardiac surgery and millions will suffer a major cardiovascular complication. Bleeding in the perioperative setting can lead to immediate death or trigger a cascade of events associated with major complications. TXA has the potential to prevent perioperative bleeding but a trial is needed to demonstrate such a benefit and to establish safety.

The effects of noncardiac surgery and anesthesia on patient hemodynamics (e.g., blood pressure) can play a fundamental role in the pathophysiology of perioperative cardiovascular complications. Whether a hypotension-avoidance or a hypertension-avoidance BP management strategy in the perioperative setting will prevent major cardiovascular complications is another fundamental question that requires an answer.

POISE-3 will answer two crucial management questions and influence future perioperative practices around the world.

## Trial progress

This paper is based on the most recent version of the study protocol (i.e., v7.0, 2021-07-22). The first patient was randomized on June 27, 2018. The DMC undertook the planned interim analyses and recommended continuation of the trial. This study protocol was submitted when recruitment in the study was completed but before completion of last patient/last visit. A few protocol amendments have been implemented during the course of the trial with the most recent version of the protocol here reported being formally finalized close to the end of the recruitment. There was consensus among the POISE-3 investigators to publish the study protocol before closure of the study database but only in its definitive version, i.e., the one that will inform the report of the study results. Recruitment has involved 106 centers across 22 countries.

## Supplementary Information

**Additional file 1.** POISE-3 SPIRIT checklist.

**Additional file 2.** POISE-3 detailed inclusion criteria including definitions.

**Additional file 3.** Rationale for POISE-3 study dosing regimen of tranexamic acid.

**Additional file 4.** POISE-3 App for the blood pressure management factorial.

**Additional file 5.** POISE-3 tertiary outcomes.

**Additional file 6.** POISE-3 outcome definitions.

**Additional file 7.** POISE-3 interim analyses.

**Additional file 8.** POISE-3 organizational structure and oversight of trial conduct.

**Additional file 9.** POISE-3 informed consent form template (English).

**Additional file 10.** List of investigators and committees.

## Data Availability

The PHRI is the sponsor of this trial. The PHRI believes the dissemination of clinical research results is vital and sharing of data is important. PHRI prioritizes access to data analyses to researchers who have worked on the trial for a significant duration, have played substantial roles, and have participated in raising the funds to conduct the trial. PHRI balances the length of the research study, and the intellectual and financial investments that made it possible with the need to allow wider access to the data collected. Data will be disclosed only upon request and approval of the proposed use of the data by a Review Committee. Data will be available to the journal for evaluation of reported analyses. Data requests from other non-POISE-3 investigators will not be considered until 5 years after the close out of the trial.

## Abbreviations

ACEI: Angiotensin-converting enzyme inhibitor
ARB: Angiotensin-receptor blocker
BIMS: Bleeding independently associated with mortality after noncardiac surgery
BP: Blood pressure
DMC: Data Monitoring Committee
eGFR: Estimated glomerular filtration rate
MINS: Myocardial injury after noncardiac surgery
PHRI: Population Health Research Institute
POISE: Perioperative Ischemic Evaluation Study
RCT: Randomized controlled trial
SAE: Serious adverse event
SBP: Systolic blood pressure
SUSAR: Suspected unexpected serious adverse reactions
TXA: Tranexamic acid
VISION: Vascular events In noncardiac Surgery patIents cOhort evaluatioN
