# Supplementary material for: Rationale and design of the PeriOperative ISchemic Evaluation-3 (POISE-3): a randomized controlled trial evaluating tranexamic acid and a strategy to minimize hypotension in noncardiac surgery
Source: Trials. 2022 Jan 31;23:101. doi: 10.1186/s13063-021-05992-1 (PMC8805242; doi:10.1186/s13063-021-05992-1)
Supplement: Supplementary file 2 — Additional file 2. POISE-3 detailed inclusion criteria including definitions. [file 13063_2021_5992_MOESM2_ESM.docx]

# POISE-3 detailed inclusion criteria with definitions

Patients need to meet the following criteria for inclusion:

1. Undergoing noncardiac surgery;
2. ≥ 45 years of age;
3. Expected to require at least one overnight hospital admission after surgery;
4. Provide written informed consent to participate in the POISE-3 Trial, AND
5. Fulfill ≥1 of the following 6 criteria (A-F):
   1. N-terminal pro–B-type natriuretic peptide (NT-proBNP) ≥200 ng/L
   2. History of coronary artery disease as defined by any one of the following 7 criteria:
      1. History of angina,
      2. History of myocardial infarction or acute coronary syndrome,
      3. History of a regional cardiac wall motion abnormality on echocardiography or a segmental fixed defect on radionuclide imaging,
      4. History of a radionuclide exercise, echocardiographic exercise, or pharmacological cardiovascular stress test demonstrating cardiac ischemia,
      5. History of a coronary angiographic or CT coronary angiographic evidence of atherosclerotic stenosis ≥ 50% of the diameter of any coronary artery,
      6. ECG with pathological Q waves in two contiguous leads, OR
      7. Previous coronary artery revascularization, i.e. percutaneous coronary intervention (PCI) or coronary artery bypass graft surgery (CABG)
   3. History of peripheral arterial disease as defined by a physician diagnosis of a current, or prior history of any one of the following 4 criteria:
      1. Intermittent claudication
      2. Vascular surgery for atherosclerotic disease
      3. An ankle/arm systolic blood pressure ratio <0.90 in either leg at rest, OR
      4. Angiographic or doppler study demonstrating >70% stenosis in a noncardiac artery
   4. History of stroke as defined by any one of the following 2 criteria
      1. A physician diagnosis of stroke, OR
      2. CT or MRI evidence of a prior stroke
   5. Undergoing major vascular surgery defined as all vascular surgery except arteriovenous shunt, vein stripping procedures, carotid endarterectomies, and endovascular abdominal aortic aneurysm repair (EVAR); OR
   6. Any 3 of 9 risk criteria:
      1. Undergoing major surgery defined as intraperitoneal, intrathoracic, retroperitoneal or major orthopedic surgery (i.e., hip arthroplasty, internal fixation of hip or femur, pelvic arthroplasty, knee arthroplasty, above-knee amputation or amputation below the knee but above the foot);
      2. History of congestive heart failure defined as a physician diagnosis of a current or prior episode of congestive heart failure OR prior radiographic evidence of vascular redistribution, interstitial pulmonary edema, or frank alveolar pulmonary edema;
      3. History of a transient ischemic attack;
      4. Diabetes and currently taking an oral hypoglycemic agent or insulin;
      5. Age >70 years;
      6. History of hypertension;
      7. Serum creatinine > 175 µmol/L (> 2.0 mg/dl) based on most recent before randomization;
      8. History of smoking within 2 years of surgery;
      9. Undergoing emergent/urgent surgery defined as surgery that a surgeon schedules to go to the operating room within 48 hours of an acute presentation to the hospital.
