# Supplementary material for: Rationale and design of the PeriOperative ISchemic Evaluation-3 (POISE-3): a randomized controlled trial evaluating tranexamic acid and a strategy to minimize hypotension in noncardiac surgery
Source: Trials. 2022 Jan 31;23:101. doi: 10.1186/s13063-021-05992-1 (PMC8805242; doi:10.1186/s13063-021-05992-1)
Supplement: Supplementary file 3 — Additional file 3. Rationale for POISE-3 study dosing regimen of tranexamic acid. [file 13063_2021_5992_MOESM3_ESM.docx]

# Rationale for POISE-3 study dosing regimen of tranexamic acid

In POISE-3, patients are randomized to receive two intravenous doses of either 1 g of tranexamic acid (TXA) or matching placebo (i.e., equivalent volume of 0.9% normal saline), as a bolus or 10-minute infusion. The first dose is given at the beginning of surgery (i.e., within 20 minutes preceding the anticipated skin incision), and the second at the end of surgery (i.e., at wound closure). We selected this dosing regimen based on the available literature. A meta-analysis of RCTs evaluating intravenous TXA in orthopedic surgery showed that dosing regimens lower than 30 mg/kg are effective in reducing blood transfusion.^1^ The meta-analysis also suggested additional efficacy of regimens including one bolus followed by a repeated bolus or continuous infusion, compared with regimens including only one single bolus.^1^ In the by-dose sub-analysis of a cohort study on 872,416 patients having total knee or hip arthroplasty in 510 hospitals, the 2 g dose was associated with the best effectiveness and safety profile compared with ≤1 g or ≥3 g dosing regimens.^2^

***References***

1. Zufferey P, Merquiol F, Laporte S, et al. Do antifibrinolytics reduce allogeneic blood transfusion in orthopedic surgery? *Anesthesiology* 2006;105(5):1034-46.

2. Poeran J, Rasul R, Suzuki S, et al. Tranexamic acid use and postoperative outcomes in patients undergoing total hip or knee arthroplasty in the United States: retrospective analysis of effectiveness and safety. *BMJ* 2014;349:g4829. doi: 10.1136/bmj.g4829
