# Supplementary material for: Rationale and design of the PeriOperative ISchemic Evaluation-3 (POISE-3): a randomized controlled trial evaluating tranexamic acid and a strategy to minimize hypotension in noncardiac surgery
Source: Trials. 2022 Jan 31;23:101. doi: 10.1186/s13063-021-05992-1 (PMC8805242; doi:10.1186/s13063-021-05992-1)

# POISE-3 App for the blood pressure management factorial

The POISE-3 National Leader in Australia and his team developed a POISE-3 iOS App for the blood pressure (BP) management factorial. The App is free to download through the App Store but requires a password to unlock, which is provided only to POISE-3 investigators. It includes information about the trial, has a tool for determining patient eligibility, and assists with the creation of a treatment plan for the BP management factorial depending on the randomization group.

**Screenshot 1: App menu page**


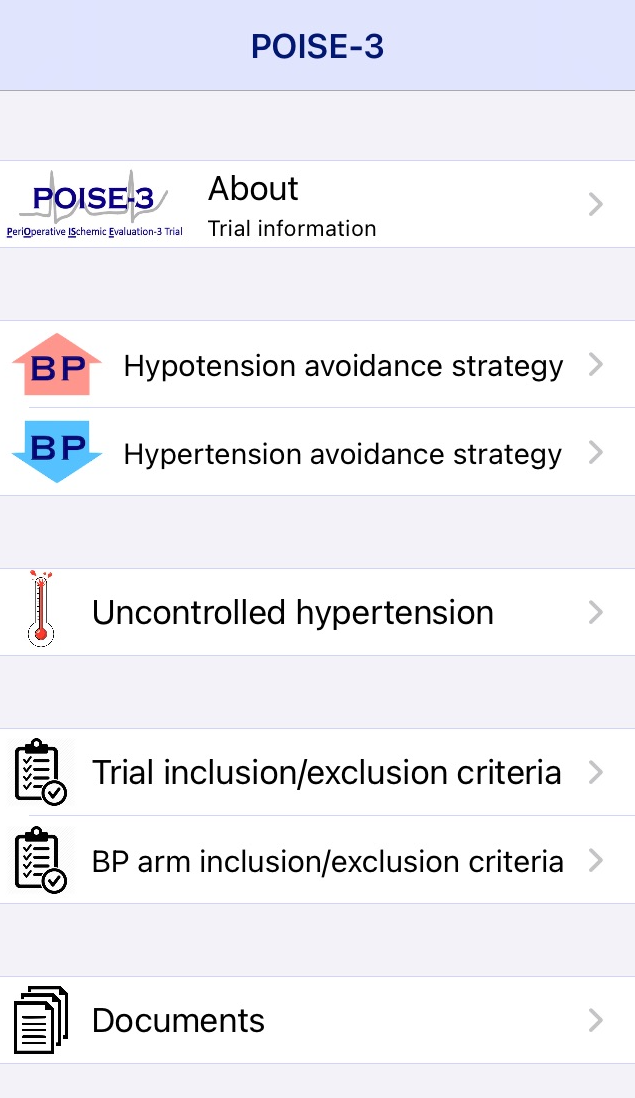


**Screenshot 2: Search tool for antihypertensive medications**


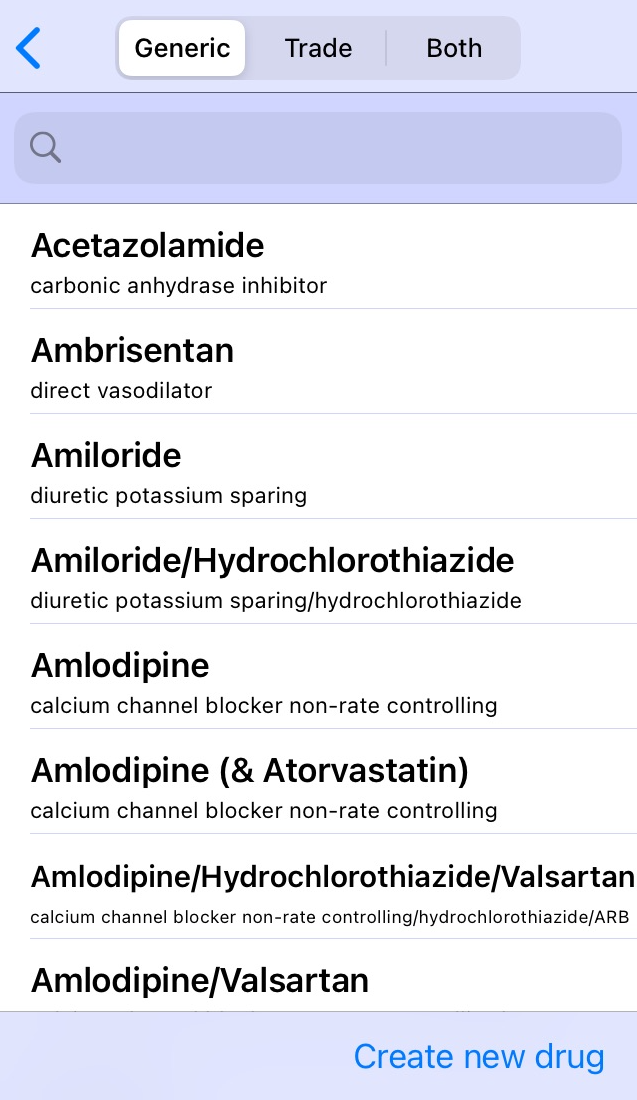


**Screenshot 3: Example of treatment plan for a patient on chronic treatment with amlodipine and ramipril, randomized to the hypotension-avoidance strategy**


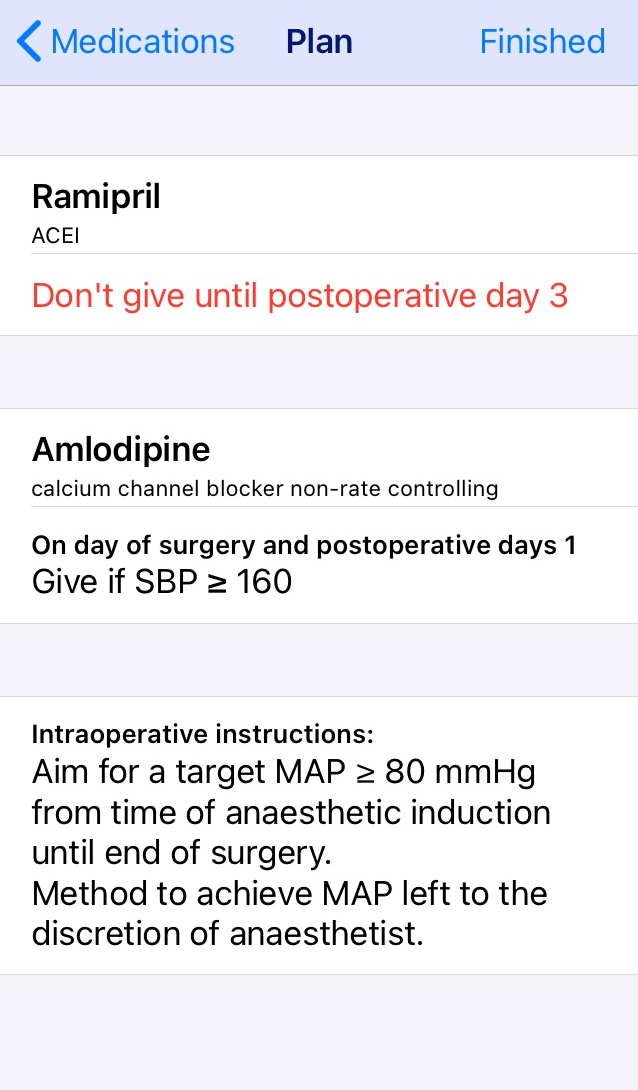

Supplement: Supplementary file 4 — Additional file 4. POISE-3 App for the blood pressure management factorial. [file 13063_2021_5992_MOESM4_ESM.docx]
