# Supplementary material for: Rationale and design of the PeriOperative ISchemic Evaluation-3 (POISE-3): a randomized controlled trial evaluating tranexamic acid and a strategy to minimize hypotension in noncardiac surgery
Source: Trials. 2022 Jan 31;23:101. doi: 10.1186/s13063-021-05992-1 (PMC8805242; doi:10.1186/s13063-021-05992-1)
Supplement: Supplementary file 5 — Additional file 5. POISE-3 tertiary outcomes. [file 13063_2021_5992_MOESM5_ESM.docx]

# POISE-3 tertiary outcomes

The tertiary outcomes for the tranexamic acid (TXA) trial at 30 days after randomization include: all-cause mortality; vascular mortality; International Society on Thrombosis and Haemostasis (ISTH) major bleeding; non-hemorragic stroke, peripheral arterial thrombosis; symptomatic proximal venous thromboembolism; transfusions (i.e. proportion of patients transfused); hemorrhagic stroke; cardiac revascularization; amputation; symptomatic pulmonary embolism; symptomatic proximal deep vein thrombosis; any symptomatic or asymptomatic proximal venous thromboembolism; acute kidney injury; new renal replacement therapy; re-hospitalization for vascular reasons; seizures; infection/sepsis; length of hospital stay; and number of days alive and at home.

The tertiary outcomes at 30 days after randomization for the blood pressure (BP) management factorial include: non-fatal cardiac arrest; hemorrhagic stroke; non-hemorrhagic stroke; acute kidney injury; new renal replacement therapy; acute congestive heart failure; new clinically important atrial fibrillation; sepsis; cancellation/postponement of surgery on the day of surgery due to BP concerns; length of hospital stay; and number of days alive and at home.

The tertiary outcomes at 1 year after randomization for the TXA trial include: all-cause mortality; vascular mortality; myocardial infarction; cardiac arrest; hemorrhagic stroke; non-hemorrhagic stroke; peripheral arterial thrombosis; amputation; symptomatic pulmonary embolism; symptomatic proximal deep vein thrombosis; symptomatic proximal venous thromboembolism; any symptomatic or asymptomatic proximal venous thromboembolism; new renal replacement therapy; re-hospitalization for vascular reasons; seizures; infection/sepsis; and disability (based on 12-item [WHODAS 2.0]).

The tertiary outcomes at 1 year after randomization for the BP management factorial include: all-cause mortality; vascular mortality; myocardial infarction; cardiac arrest; hemorrhagic stroke; non-hemorrhagic stroke; new renal replacement therapy; acute congestive heart failure, sepsis; and disability (based on 12-item [WHODAS 2.0]).

The analyses on the effects of the study interventions on renal outcomes are part of a substudy and will be presented in a separate paper.
