# Supplementary material for: Rationale and design of the PeriOperative ISchemic Evaluation-3 (POISE-3): a randomized controlled trial evaluating tranexamic acid and a strategy to minimize hypotension in noncardiac surgery
Source: Trials. 2022 Jan 31;23:101. doi: 10.1186/s13063-021-05992-1 (PMC8805242; doi:10.1186/s13063-021-05992-1)
Supplement: Supplementary file 6 — Additional file 6. POISE-3 outcome definitions. [file 13063_2021_5992_MOESM6_ESM.docx]

# POISE-3 outcome definitions

1. **Sub-Classification of Death**

Vascular death is defined as any death with a vascular cause and includes those deaths following a myocardial infarction, cardiac arrest, stroke, cardiac revascularization procedure (i.e., percutaneous coronary intervention [PCI] or coronary artery bypass graft [CABG] surgery), pulmonary embolus, hemorrhage, or deaths due to an unknown cause. Non-vascular death is defined as any death due to a clearly documented non-vascular cause (e.g. trauma, infection, malignancy).

1. **Myocardial Injury after Noncardiac Surgery (MINS)**

MINS is defined as any myocardial infarction (as defined below), and any elevated troponin (higher than the local lab threshold) judged to be due to myocardial ischemia (i.e., without evidence of a non-ischemic etiology [e.g., chronic elevation, pulmonary embolism, sepsis, cardioversion]) that occurred within the first 30 days after the initiation of surgery. The only exceptions to the definition of an elevated troponin will be to use a higher threshold for troponin T (TnT) of ≥30 ng/L, and for high-sensitivity troponin T (hsTnT) of 20 to <65 ng/L with an absolute change of at least 5 ng/L or an hsTnT level ≥65 ng/L.  These threshold for TnT and hsTnT are based upon data from a large international prospective perioperative cohort study that established troponin thresholds that were independently associated with 30-day mortality after non-cardiac surgery.

1. **Myocardial Infarction**

If the diagnostic criteria for myocardial infarction includes an elevated troponin, then the definition of MINS must be met to fulfill the diagnostic criteria for myocardial infarction. The diagnosis of myocardial infarction requires any one of the following criteria.

- - - 1. Detection of a rise or fall of a cardiac biomarker (preferably troponin) with at least one value above the 99^th^ percentile of the upper reference limit (URL) together with evidence of myocardial ischemia with at least one of the following:
         1. ischemic signs or symptoms (i.e., chest, arm, neck, or jaw discomfort; shortness of breath, pulmonary edema);
         2. development of pathologic Q waves present in any two contiguous leads that are ≥ 30 milliseconds;
         3. new or presumed ECG changes indicative of ischemia (i.e., ST segment elevation [≥ 2 mm in leads V_1_, V_2_, or V_3_ OR ≥ 1 mm in the other leads], ST segment depression [≥ 1 mm], or symmetric inversion of T waves ≥ 1 mm) in at least two contiguous leads;
         4. new LBBB; or
         5. new cardiac wall motion abnormality on echocardiography or new fixed defect on radionuclide imaging
         6. identification of intracoronary thrombus on angiography or autopsy
      2. Cardiac death, with symptoms suggestive of myocardial ischemia and presumed new ischemic ECG changes or new LBBB, but death occurred before cardiac biomarkers were obtained, or before cardiac biomarker values would be increased.
      3. Percutaneous coronary intervention (PCI) related myocardial infarction is defined by elevation of a troponin value (>5 x 99th percentile URL) in patients with a normal baseline troponin value (≤99th percentile URL) or a rise of a troponin measurement >20% if the baseline values are elevated and are stable or falling. In addition, either (i) symptoms suggestive of myocardial ischemia or (ii) new ischemic ECG changes or (iii) angiographic findings consistent with a procedural complication or (iv) imaging demonstration of new loss of viable myocardium or new regional wall motion abnormality are required.

- - - 1. Stent thrombosis associated with myocardial infarction when detected by coronary angiography or autopsy in the setting of myocardial ischemia and with a rise and/or fall of cardiac biomarker values with at least one of value above the 99th percentile URL.
      2. Coronary artery bypass grafting (CABG) related myocardial infarction is defined by elevation of cardiac biomarker values (>10 x 99th percentile URL) in patients with a normal baseline troponin value (≤99th percentile URL). In addition, either (i) new pathological Q waves or new LBBB, or (ii) angiographic documented new graft or new native coronary artery occlusion, or (iii) imaging evidence of new loss of viable myocardium or new regional wall motion abnormality.
      3. For patients who are believed to have suffered a myocardial infarction within 28 days of a MINS event or within 28 days of a prior myocardial infarction, the following criterion for myocardial infarction is required:

Detection of a rise or fall of a cardiac biomarker (preferably troponin) with at least one value above the 99^th^ percentile of the upper reference limit (URL) and 20% higher than the last troponin measurement related to the preceding event together with evidence of myocardial ischemia with at least one of the following:

- - - - 1. ischemic signs or symptoms (i.e., chest, arm, neck, or jaw discomfort; shortness of breath, pulmonary edema);
        2. development of pathologic Q waves present in any two contiguous leads that are > 30 milliseconds;
        3. new or presumed new ECG changes indicative of ischemia (i.e., ST segment elevation [> 2 mm in leads V_1_, V_2_, or V_3_ OR > 1 mm in the other leads], ST segment depression [> 1 mm], or symmetric inversion of T waves > 1 mm) in at least two contiguous leads;
        4. new LBBB; or
        5. new cardiac wall motion abnormality on echocardiography or new fixed defect on radionuclide imaging
        6. identification of intracoronary thrombus on angiography or autopsy

1. **Stroke**

Stroke is defined as a new focal neurological deficit thought to be vascular in origin with signs or symptoms lasting more than 24 hours or leading to death. Stroke will be sub-classified into hemorrhagic and non-hemorrhagic stroke. Non-hemorrhagic stroke will sub-classified into ischemic, ischemic with secondary transformation, or stroke of uncertain classification. Hemorrhagic stroke will be sub-classified into primary intracerebral hemorrhage and primary subarachnoid hemorrhage.

##### ***Ischemic stroke***: focal brain infarction caused by an arterial (or rarely venous) obstruction and as documented by CT/MRI that is normal or shows an infarct in the clinically expected area.

1. ***Secondary hemorrhagic transformation of ischemic stroke***: hemorrhagic transformation of ischemic stroke may be symptomatic or asymptomatic.
2. Symptomatic transformation of ischemic stroke is defined as a hematoma occupying 30% or more of the infarcted tissue associated with a significant neurologic deterioration (consistent with a decrease of 4 points in the NIHSS) compared to immediately before the worsening and an absence of an alternative explanation for deterioration.
3. Asymptomatic transformation of ischemic stroke is defined as a hemorrhagic transformation not meeting the criteria for symptomatic transformation.
4. ***Undetermined stroke***: definite stroke that does not meet the criteria for ischemic or hemorrhagic stroke because CT scan or MRI are not done and there are no autopsy data. Rarely it cannot be determined with confidence whether the stroke was ischemic vs. hemorrhagic, even after review of CT/MRI images (e.g., primary intracerebral hemorrhage vs. severe hemorrhagic transformation); these stroke events will be classified as undetermined.
5. ***Hemorrhagic stroke***: hemorrhagic stroke requires neuroimaging or autopsy confirmation and includes two subcategories: primary intracerebral hemorrhage (intraparenchymal or intraventricular) and primary subarachnoid hemorrhage. Intracranial bleeding caused by head trauma, bleeding associated with tumors, hemorrhagic transformation of ischemic stroke and subdural/epidural hematomas are not considered as hemorrhagic strokes (but these will be counted separately as major hemorrhages). Microbleeds are not considered intracranial hemorrhage.
6. Primary intracerebral hemorrhage: These are symptomatic hemorrhagic strokes with CT/MRI or autopsy evidence of bleeding into the substance of the brain or ventricular spaces. Large or superficial intracerebral hemorrhages often are associated with minor amounts of subarachnoid hemorrhage, but these should be classified as intracerebral hemorrhages. Does not include secondary hemorrhage into cerebral infarct (i.e. hemorrhagic transformation which is defined separately), or intracerebral bleeding (i.e. contusions) due to trauma, or microbleeds detected by MRI.
7. Primary subarachnoid hemorrhage: Typical clinical syndrome of sudden onset headache, with or without focal signs (subarachnoid hemorrhage may not have focal deficits), and CT or cerebrospinal fluid evidence of bleeding primarily into the subarachnoid space. Subarachnoid bleeding due to ruptured intracranial aneurysms and vascular malformation are counted as hemorrhagic strokes, but traumatic subarachnoid hemorrhage is not.
8. **Symptomatic Proximal Venous Thromboembolism**

Venous thromboembolism that includes symptomatic pulmonary embolism or symptomatic proximal deep vein thrombosis

1. **Symptomatic Pulmonary Embolism (PE)**

The diagnosis of symptomatic PE requires symptoms (e.g., dyspnea, pleuritic chest pain) or signs (e.g., hypoxia, increased work of breathing) and any one of the following:

1. A high probability ventilation/perfusion lung scan,
2. An intraluminal filling defect of segmental or larger artery on a helical CT scan,
3. An intraluminal filling defect on pulmonary angiography, or
4. A positive diagnostic test for DVT (e.g., positive compression ultrasound) and one of the following:

non-diagnostic (i.e., low or intermediate probability) ventilation/perfusion lung scan, or

non-diagnostic (i.e., subsegmental defects or technically inadequate study) helical CT scan

1. **Symptomatic Proximal Deep Venous Thrombosis (DVT)**

The diagnosis of symptomatic proximal deep venous thrombosis requires:

1. symptoms or signs that suggest DVT (e.g., leg pain or swelling),
2. thrombosis involving the popliteal vein or more proximal veins for leg DVT OR axillary or more proximal veins for arm DVTs

Any of the following defines evidence of vein thrombosis:

- - 1. a persistent intraluminal filling defect on contrast venography (including on computed tomography),
    2. noncompressibility of one or more venous segments on B mode compression ultrasonography, or
    3. A clearly defined intraluminal filling defect on doppler imaging in a vein that cannot have compressibility assessed (e.g., iliac, inferior vena cava, subclavian).

1. **Nonfatal cardiac arrest**

Nonfatal cardiac arrest is defined as successful resuscitation from either documented or presumed ventricular fibrillation, sustained ventricular tachycardia, asystole, or pulseless electrical activity requiring cardiopulmonary resuscitation, pharmacological therapy, or cardiac defibrillation.

1. **MINS not fulfilling the universal definition of MI**

Any elevated troponin (higher than the local lab threshold) judged to be due to myocardial ischemia (i.e. without evidence of a non-ischemic etiology, e.g. chronic elevation, pulmonary embolism, sepsis, cardioversion, others) that occurred with the first 30 days after surgery, and not fulfilling the definition of MI (as defined above).

1. **Cardiac Revascularization Procedures**

Cardiac revascularization procedures include PCI and CABG surgery.

1. **Amputation**

Amputation is defined as an amputation procedure, or auto amputation subsequent to the initial surgery.

1. **Peripheral Arterial Thrombosis**

We will consider a peripheral arterial thrombosis to have occurred where there is clear evidence of abrupt occlusion of a peripheral artery (i.e., not a stroke related to an intracranial artery or myocardial infarction) consistent with either an acute local thrombotic event or a peripheral arterial embolism.  To fulfill this definition we require at least one of the following objective findings of peripheral arterial thrombosis:

1. Surgical report indicating evidence of arterial thrombosis/ peripheral arterial embolism,
2. Pathological specimen demonstrating arterial thrombosis/ peripheral arterial embolism,
3. Imaging evidence consistent with arterial thrombosis/ peripheral arterial embolism, or
4. Autopsy reports documenting arterial thrombosis/ peripheral arterial embolism
5. **Life-threatening bleeding**

Life-threatening bleeding is bleeding that is fatal, or leads to: significant hypotension that requires inotrope therapy, urgent (within 24 hours) surgery (other than superficial vascular repair), or intracranial hemorrhage.

1. **Major bleeding**

Major bleeding is defined as bleeding that is not specified under “life- threatening bleeding” above, and results in a postoperative hemoglobin ≤ 70 g/L; or the patient receives a transfusion of ≥ 1 unit of red blood cells; or leads to one of the following interventions (i.e., embolization, superficial vascular repair, nasal packing).

1. **Critical organ bleeding**

A critical organ bleeding event was bleeding that was intracranial, intraocular, intraspinal, pericardial, retroperitoneal, or intramuscular with compartment syndrome.

1. **ISTH major bleeding**

ISTH major bleeding is a bleed that meets any of the following:

1. Fatal bleeding,
2. Bleeding that is symptomatic and occurs in a critical area or organ, such as intracranial, intraspinal, intraocular, retroperitoneal, pericardial, in a non-operated joint, or intramuscular with compartment syndrome, assessed in consultation with the surgeon, and/or,
3. Extrasurgical site bleeding causing a fall in hemoglobin level of 20 g/L (1.24 mmol/L) or more, or leading to transfusion of two or more units of whole blood or red cells, with temporal association within 24–48 h to the bleeding
4. Surgical site bleeding that requires a second intervention - open, arthroscopic, endovascular - or a hemarthrosis of sufficient size as to interfere with rehabilitation by delaying mobilization or delayed wound healing, resulting in prolonged hospitalization or a deep wound infection, or
5. Surgical site bleeding that is unexpected and prolonged and/ or sufficiently large to cause hemodynamic instability, as assessed by the surgeon. There should be an associate fall in hemoglobin level of 20 g/L (1.24 mmol/L), or transfusion, indicated by the bleeding, of at least two units of whole blood or red cells, with temporal association within 24 h to the bleeding.
6. **Bleeding Independently Associated with Mortality after noncardiac Surgery (BIMS)**

BIMS is a bleeding meeting any of the following 3 criteria:

1. Leading to a postoperative hemoglobin <70 g/L
2. Requiring transfusion of one or more units of red blood cells
3. Judged to be the immediate cause of death
4. **New clinically important atrial fibrillation**

The definition of clinically important atrial fibrillation requires the documentation of atrial fibrillation or atrial flutter of any duration on an ECG or rhythm strip, which results in angina congestive heart failure, symptomatic hypotension, or requires treatment with a rate controlling drug, antiarrhythmic drug, or electrical cardioversion.

1. **Acute congestive heart failure**

The definition of congestive heart failure requires at least one of the following clinical signs (i.e. any of the following signs: elevated jugular venous pressure, respiratory rales/crackles, crepitations, or presence of S3) and at least one of the following:

1. Radiographic findings (i.e., vascular redistribution, interstitial pulmonary edema, or frank alveolar pulmonary edema) OR
2. Heart failure treatment implemented with diuretics with documented clinical improvement.
3. **Acute kidney injury**

An acute kidney injury is defined as an increase in serum creatinine concentration from the preoperative (pre-randomization) concentration by either an increase of ≥26.5 μmol/L (≥0.3 mg/dL)) within 48 hours of surgery or an increase of 50% or greater within 7 days of surgery.

1. **New requirement of renal replacement therapy (dialysis)**

Dialysis is defined as the use of a hemodialysis machine or peritoneal dialysis apparatus.

1. **Infection and Sepsis**

Infection is defined as a pathologic process caused by the invasion of normally sterile tissue or fluid or body cavity by pathogenic or potentially pathogenic organisms.

The Third International Consensus Definitions Task Force defines sepsis as a “life-threatening organ dysfunction due to a dysregulated host response to infection.” Based on the Third International Consensus Definitions for Sepsis and Septic Shock (Sepsis-3) criteria, sepsis will require a quick Sequential Organ Failure Assessment (qSOFA) Score ≥2 points due to infection. The qSOFA includes the following items and scoring system:

1. Glasgow Coma Scale (GCS) score of 13 or less (1 point)
2. systolic blood pressure of 100 mm Hg or less (1 point), and
3. respiratory rate of 22 breaths/min or more (1 point).
4. **Rehospitalization for Vascular Reasons**

Re-hospitalization for vascular reasons is defined as re-hospitalization for myocardial infarction, non-fatal cardiac arrest, stroke, congestive heart failure, ischemic symptoms with ST or T wave changes on an ECG, cardiac arrhythmia, cardiac revascularization procedure, amputation, peripheral arterial thrombosis, DVT, pulmonary embolus, any vascular surgery, or bleeding.

1. **Seizure**

Seizure is defined as the abrupt onset of focal or generalized experiential, motor, sensory or cognitive phenomena, in absence of another etiology for the event (e.g., movement or psychiatric disorder).

1. **Disability**

The health and disability will be measured using the 12-item version of the WHODAS 2.0, which assesses the level of functioning in 6 domains of life, i.e., *cognition*, *mobility*, *self-care*, *getting along*, *life activities*, and *participation*. WHODAS 2.0 will be administered in person or over the telephone, at baseline, 30 days and 1 year of follow-up. At each time point, the score will be calculated using the simple approach that consists in summing the scores assigned to each item. Disability is defined as a persistent (≥6 months) reduction in health status as measured by a 12-item version of WHODAS score of at least 12 points, reflecting a disability level of at least 25% and being the threshold point between “disabled” and “not disabled” as per WHO guidelines”.
