# Supplementary material for: Rationale and design of the PeriOperative ISchemic Evaluation-3 (POISE-3): a randomized controlled trial evaluating tranexamic acid and a strategy to minimize hypotension in noncardiac surgery
Source: Trials. 2022 Jan 31;23:101. doi: 10.1186/s13063-021-05992-1 (PMC8805242; doi:10.1186/s13063-021-05992-1)
Supplement: Supplementary file 7 — Additional file 7. POISE-3 interim analyses. [file 13063_2021_5992_MOESM7_ESM.docx]

# POISE-3 interim analyses

For the tranexamic acid (TXA) trial, three interim analyses based on the primary safety outcome will occur when 25%, 50% and 75% of the 30-day data are available. The Data Monitoring Committee (DMC) will employ the modified Haybittle-Peto rule of 3.5 standard deviations (SDs) (α = 0.00047).^1 2^ To trigger discussion about stopping the trial early for harm, the point estimate of the hazard ratio (HR) for TXA versus placebo needs to exceed 1.0 by more than 3.5 SDs at any of the interim analyses.

Two interim analyses for efficacy of TXA will occur when 50% and 75% of the 30-day data are available. They will be based on a net risk-benefit outcome defined as the composite of vascular death, bleeding (i.e., non-fatal life-threatening, major, or critical organ), myocardial injury after noncardiac surgery (MINS), stroke, peripheral arterial thrombosis, and symptomatic venous thromboembolism. The DMC will employ the modified Haybittle-Peto rules^1 2^ of 4 SDs (α = 0.000067) for the interim analyses on the 50% subset, and of 3.5 SDs (α = 0.00047) for the 75% subset analysis. For the results of the interim analyses to be considered significant to trigger discussions about stopping the trial for greater than expected efficacy, these predefined boundaries will have to be exceeded in at least 2 consecutive analyses, 3 or more months apart.

For the blood pressure (BP) management factorial, three interim efficacy analyses based on the primary outcome will occur when 25%, 50% and 75% of the 30-day data are available. The DMC will employ the modified Haybittle-Peto rule of 4 SDs (α = 0.000067) for analyses in the first half of the trial (including the second planned interim analysis) and 3.5 SDs (α = 0.00047) for all analyses in the second half. For a finding to be considered significant, these predefined boundaries will have to be exceeded in at least 2 consecutive analyses, 3 or more months apart.

The DMC will monitor for an adverse impact of the BP perioperative hypotension- or hypertension- avoidance managements on mortality. For this analysis, a 3.5 SDs excess in the first half and a 2.6 SDs (α = 0.00932) excess in the second half of the trial would trigger discussions about stopping for harm.

The α-level for the final analysis will remain the conventional two-sided α = 0.05 (or one-sided 97.5% CI for the primary safety outcome for the TXA trial) given the infrequent interim analyses, their extremely low α levels, and the requirement for confirmation with subsequent analyses.

At any time during the trial if safety concerns arise the DMC chairperson will assemble a formal meeting of the full committee. The DMC will make their recommendations to the Project Office Operations Committee after considering all the available data and any external data from relevant studies. If a recommendation for termination is being considered, the DMC will invite the Project Office Operations Committee to explore all possibilities before a decision is made. A detailed Charter governs the activities of the DMC. The DMC has members with expertise in clinical trials, perioperative medicine, and biostatistics.

***References***

1. Haybittle JL. Repeated assessment of results in clinical trials of cancer treatment. *Br J Radiol* 1971;44(526):793-7. doi: 10.1259/0007-1285-44-526-793

2. Peto R, Pike MC, Armitage P, et al. Design and analysis of randomized clinical trials requiring prolonged observation of each patient. I. Introduction and design. *Br J Cancer* 1976;34(6):585-612.
