# Supplementary material for: Rationale and design of the PeriOperative ISchemic Evaluation-3 (POISE-3): a randomized controlled trial evaluating tranexamic acid and a strategy to minimize hypotension in noncardiac surgery
Source: Trials. 2022 Jan 31;23:101. doi: 10.1186/s13063-021-05992-1 (PMC8805242; doi:10.1186/s13063-021-05992-1)
Supplement: Supplementary file 8 — Additional file 8. POISE-3 organizational structure and oversight of trial conduct. [file 13063_2021_5992_MOESM8_ESM.docx]

## POISE-3 Organizational Structure

**Trial hospitals**

**(Research personnel)**

**Project Office**

**Operations Committee**

**Project Office**

**(Trial coordinator,**

**Project Officer,**

**Principal Investigator)**

**National Leaders**

**Event Adjudication Committee**

**Sub-study and Publication Committee**

**Centre Principal Investigator**

**Steering Committee**

**Data Monitoring Committee**

## Oversight of study conduct

The Project Office Operations Committee (POOC) has met quarterly to review trial progress and all pertinent issues related to the conduct of POISE-3. The International Operations Committee (IOC), to which the POOC directly reports, has held biannual calls to review the progress of the trial, discuss international trial issues, and strategize to ensure the successful conduct and completion of POISE-3. The IOC reports to the Steering Committee (SC), which includes the Principle Investigator, members with expertise in trial design and conduct, and one National Leader (NL) for each participating country. The SC has met at least annually. At the SC meetings, each NL has reported to the SC regarding the country’s progress in POISE-3, goals for the coming year, and any issues that require input.

## 
