# Supplementary material for: Rationale and design of the PeriOperative ISchemic Evaluation-3 (POISE-3): a randomized controlled trial evaluating tranexamic acid and a strategy to minimize hypotension in noncardiac surgery
Source: Trials. 2022 Jan 31;23:101. doi: 10.1186/s13063-021-05992-1 (PMC8805242; doi:10.1186/s13063-021-05992-1)
Supplement: Supplementary file 9 — Additional file 9. POISE-3 informed consent form template (English). [file 13063_2021_5992_MOESM9_ESM.pdf]

POISE-3

PeriOperative ISchemic Evaluation-3 Trial

## PATIENT INFORMATION and INFORMED CONSENT

**Study Title:** PeriOperative ISchemic Evaluation-3 (POISE-3) Trial

**Sponsor:** Population Health Research Institute, Hamilton, Canada

**Site Principal Investigator:**

**Investigator Site:**

## PATIENT INFORMATION

You are being invited to take part in this research study because you are undergoing surgery, and are at risk of having a bleeding event, or a complication related to the heart or blood vessels. It is important you carefully read and understand the following information about the study. Please ask as many questions as you like. Reading and signing this consent form is necessary before you can participate. You will be treated with the same level of care whether or not you agree to participate. This international study is being conducted by the Population Health Research Institute (PHRI) located in Hamilton, Canada.

## BACKGROUND AND PURPOSE OF STUDY

More than 200 million adults undergo major surgery every year. Despite the benefits of surgery, about 10 million will experience a major complication. Studies have shown that bleeding is strongly associated with death and complications related to the heart or blood vessels for up to 30 days after surgery. Therefore, minimizing bleeding may help to prevent death and reduce the risk of complications after surgery.

Tranexamic acid (TXA), a drug given intravenously, is used to prevent or treat bleeding by preventing the breakdown of blood clots. TXA is a drug that has been approved by Health Canada for several years and is already in use in certain clinical setting. Some research suggests that TXA may reduce bleeding in non-heart surgery. However, this evidence is not definitive. Moreover, the safety of using TXA (compared to placebo) in surgery is still unclear. This purpose of this study is to assess the effect of TXA on bleeding and on the heart and circulatory complications.

There is evidence suggesting that high-blood pressure around the time of surgery might be associated with major complications. However, there is evidence to suggest that low-blood pressure around the time of surgery is frequent, and is associated with major complications and

death. Standard care in many centers is to continue treating patients with blood pressure medications right before and after surgery. However, we are not sure if this approach versus an approach that only gives high-blood pressure medications if the blood pressure is elevated will prevent major complications and death up to 30-days after surgery.

**STUDY OUTLINE AND INTERVENTIONS**

A total of 10,000 patients will participate in this study at around 150 hospitals in 25 countries. The study should take around 4 years to complete, and results should be known in about 4 years. Results will be available on clinicaltrials.gov.

If you agree to participate, you will be randomly assigned by an automated system to one of two treatment groups: TXA (1 g given intravenously right before surgery, 1 g given intravenously at the end of surgery) or placebo (0.9% normal saline). You will have a 50% (1 in 2) chance of receiving TXA, and a 50% (1 in 2) chance of receiving placebo. Neither you nor the study doctor/study staff will know which medication you will be receiving. However, this information will be available in case of an emergency.

In addition, if you are taking a high-blood pressure medication, you will be randomly assigned to one of two additional treatment groups: the group receiving a strategy to avoid low-blood pressure or the group receiving a strategy to avoid high-blood pressure. You will have a 50% (1 in 2) chance of being in the low-blood pressure avoidance group, and a 50% (1 in 2) chance of being in the high-blood pressure avoidance group.

The two strategies differ in the way your blood pressure medications will be managed before and after surgery, and in the level your blood pressure will be maintained during surgery. If you are assigned to the low-blood pressure avoidance strategy, your usual blood pressure medications might be held, or (some) continued based on your blood pressure on the day of surgery, and in the first 2 days after surgery. In addition, during surgery, your doctors will try to ensure that your blood pressure does not drop below a certain value.

If you are assigned to the high-blood pressure avoidance strategy, you will receive your usual blood pressure medications on the day of surgery and in the first 2 days after surgery, unless prescribed differently by other doctors. During surgery, your blood pressure will be managed as per routine practice. After the first 2 days after surgery, the study intervention will be completed, and your blood pressure and blood pressure medication(s) will be managed by your treating physician.

For all patients: Blood sample(s) will be collected in hospital for the measurement of troponins on the 1$^{st}$, 2$^{nd}$, and 3$^{rd}$ days after surgery. You may experience some pain, faintness, irritation, bruising or bleeding.

For patients in the blood pressure groups **only**: Blood sample(s) will be collected in hospital for the measurement of serum creatinine on the 1$^{st}$, 2$^{nd}$, and 3$^{rd}$ days after surgery. Any serum

creatinine measurements in the 4th-8th days after surgery, as ordered by your treating physician, will be collected.

***Follow Up***
Follow-ups will occur 30 days and 1 year after randomization by telephone. This call will last approximately 15 minutes. Study personnel will ask you questions about your health, any problems you may have had (including any hospital admission(s) you may have had), and any medications you may be taking.

For patients in the blood pressure groups **only**: The study personnel will visit each day after your surgery for the first 3 days (or until you are discharged) and they will ask you a few questions to determine if you are having any confusion.

**RISKS, SIDE EFFECTS AND/OR DISCOMFORTS**
When TXA is used for short periods of time at low doses, side effects are rare. The following side effects have been reported when it was used for longer periods of time than what you will receive in the POISE-3 trial: nausea, dizziness, vomiting, and diarrhea. High doses of the drug (higher than the amount you will receive in the study) have been associated with a low risk of seizures (below 1 of 100 patients).

For the blood pressure management part of the trial, you will be monitored as per routine practice, and your participation in the study will not prevent your doctor from providing you care that they deem necessary. On the day of surgery, and in the first 2 days post-surgery, you may receive blood pressure medications. In most cases, these will be medications which you are already currently taking, and would not pose any additional risks. The drugs used to control blood pressure in the operating room are used in routine care, and would not pose any additional risks compared to if you were not participating in this study.

***Pregnancy Risks (applicable only to women who are able to have a child)***
If you are pregnant, you cannot take part in this study. The effects of TXA on a human embryo, fetus (unborn baby) or nursing infant are not known.

***Unforeseen Risks***
All medications have a potential risk of an allergic reaction. Please tell the study doctor or staff about all problems, illnesses or injuries that happen to you during the study, even if you think they are not related to the study intervention.

**NEW FINDINGS**
If any new findings or new information is learned about this study or the study intervention(s) that may affect your willingness to continue in the study, you will be told as soon as possible.

**MEDICAL TREATMENT FOR INJURY**
In the event of an injury or illness related to one of the study interventions, you will be provided with appropriate medical treatment/care. You are not waiving your legal rights by

agreeing to participate in this study. The study doctor and the hospital still have their legal and professional responsibilities.

**POTENTIAL BENEFITS**
There is no guarantee that you will receive any benefit from taking part in this study. However, receiving the study intervention(s) may decrease your risk of having a major complication after surgery. By taking part in this study, you will contribute information about the study drug that may benefit other patients or you in the future.

**ALTERNATIVE TREATMENTS**
Choosing not to participate in the study will in no way affect your care or treatment. Currently, there is no standard of care for preventing postoperative heart problems. Your doctor will provide the best care for you.

**COSTS AND PAYMENT FOR PARTICIPATION**
You will not be charged for taking part in this study. The study drug, study visits, physical examinations and other procedures associated with the study will be at no cost to you.

**VOLUNTARY PARTICIPATION AND EARLY WITHDRAWAL**
Taking part in this research study is voluntary. You may refuse to take part at any time for any reason without penalty or loss of benefits to which you are otherwise entitled and without any effect on your future medical care. If after signing this form you refuse to receive the study intervention(s) for any reason, it does not mean that you are stopping the study. Your study physician will continue to follow you (by telephone) for the duration of the study. If you do not continue follow-up, the study physician will make the best effort to re-contact you (e.g., contacting your family or private physician, review available registries or health care databases) to determine whether you experienced any major events like death, heart attack, stroke, blood clot, or bleeding.

Even if you wish to withdraw from the study, the information about you that was collected as part of the research project between the date you signed the current form, and the date you withdraw may still be used. This is to protect the quality of the research results. However, no new information about you will be collected and used.

**CONFIDENTIALITY**
All information related to this study will remain confidential to the extent permitted by the applicable laws and/or regulations, and will not be made publicly available. **Juravinski Hospital at Hamilton Health Sciences Corporation**, relevant regulatory agencies, the investigator and members of his/her research team, representatives of the Sponsor, and the Hamilton Integrated Research Ethics Board will be granted direct access to your medical records and other records relating to this study to check that the study information is correct, to the extent permitted by applicable laws and/or regulations. By signing and dating this informed consent form, you agree to such inspection and disclosure. Any information that leaves the doctor's office will be de-identified (i.e., identifying information will be removed from the documents).

In the event of any presentation or publication regarding this study, your identity will remain confidential. Your de-identified information and results will be archived by the investigator and sponsor as per applicable laws and/or regulations.

Your personal data including date of birth, sex, ethnic background, date of death, as well as your past medical history, hospital records, clinic notes, your treatment and your response to treatment will be collected, used and stored in a de-identified format for the study. **Juravinski Hospital at Hamilton Health Sciences Corporation** will confidentially store your full name and contact details in a secure location and will not be made available to sponsor. This information will be used to contact you about the study, continue follow-up and to oversee the quality of the study.

You have the right to request information about the handling of data, what data are recorded, to require corrections of errors, transfer of data to yourself or someone else in a commonly used format, and to know who will be responsible for keeping and have access to the data.

You also have the right to file a complaint with **the Office of the Privacy Commissioner of Canada.** If you wish to apply any of your data privacy rights please contact the trial team or ethical committee using the contact details below.

**WHOM TO CONTACT**

You are free to ask questions at any time. For answers to questions relating to this research study, to report a research related injury or for information about study procedures, you may contact

Questions regarding your rights as a volunteer may be addressed to the committee that reviewed the ethical aspects of this study at **Office of the Chair of Hamilton Integrated Research Ethics Board at** Do not sign this consent form unless you have had a chance to ask questions and have received acceptable answers to all of your questions.

**POISE-3 STUDY – PATIENT INFORMED CONSENT**

I have read this consent form and my questions have been answered. By signing this consent form I will not give up any of my legal rights. I will be given a signed and dated copy of this consent form.

I agree that results of the study may be passed on to the appropriate authorities and to the Sponsor. My name and address will be kept confidential. The investigator and members of his/her research team, representatives of the sponsor, research ethics board, or local or foreign regulatory authorities will be granted direct access to my medical records and other records relating to this study to verify the information collected. My results will be archived by the investigator and sponsor as per applicable laws and/or regulations. By signing this document, I give permission for this review of my records, passing on of my information to the sponsor and the appropriate authorities, and storage of my information. I give permission for study personnel to obtain my medical information regarding my treatment received in any clinic or doctor's office or any healthcare facility that I visit (including the Niagara Health System), for research purposes, for 3 years from the date this consent is signed. ❑ **Yes** ❑ **No**

I voluntarily consent to take part in this study and I understand that I may withdraw my consent at any time. However, if I do withdraw consent to continue participation in the study, I do agree to allow the study doctor or study staff to contact me or my designated contact person at regular intervals to see how I am doing until the end of the study.

I agree to participate in the main study comparing TXA to placebo.

❑ Yes ❑ No

I agree to participate in the portion of the study comparing a low-blood pressure avoidance strategy to a high-blood pressure avoidance strategy.

❑ Yes ❑ No ❑ Not applicable

| ☐ Printed Name of Patient<br>or<br>☐ Printed Name of Patient's Legal Acceptable Representative | ☐ Signature of Patient<br>or<br>☐ Signature of Patient's Legal Acceptable Representative | Date and Time |
|---|---|---|

**STUDY PERSONNEL STATEMENT**

The person signing this consent form has had the study fully and carefully explained and has been given an opportunity to ask any questions regarding the nature, risks and benefits of the patient's participation in this research study.

| Printed Name of Person Obtaining Consent | Signature of Person Obtaining Consent | Date and Time |
|---|---|---|
