# Supplementary material for: Rationale and design of the PeriOperative ISchemic Evaluation-3 (POISE-3): a randomized controlled trial evaluating tranexamic acid and a strategy to minimize hypotension in noncardiac surgery
Source: Trials. 2022 Jan 31;23:101. doi: 10.1186/s13063-021-05992-1 (PMC8805242; doi:10.1186/s13063-021-05992-1)
Supplement: Supplementary file 10 — Additional file 10. List of investigators and committees. [file 13063_2021_5992_MOESM10_ESM.docx]

**Additional File 10. List of Investigators and Committees**

**Table of Contents**

[1 INVESTIGATORS AND COMMITTEES 2](#_Toc83326620)

[1.1 Operations Committee 2](#_Toc83326621)

[1.2 International Operations Committee 2](#_Toc83326622)

[1.3 Steering Committee 2](#_Toc83326623)

[1.4 Event Adjudication Committee 2](#_Toc83326624)

[1.5 Data and Safety Monitoring Board (DSMB) 2](#_Toc83326625)

[1.6 Project Office Staff 2](#_Toc83326626)

[1.7 Study Statisticians and Programmers 2](#_Toc83326627)

[1.8 National Leaders 2](#_Toc83326628)

[1.9 Participating Centres 3](#_Toc83326629)

# INVESTIGATORS AND COMMITTEES

## Operations Committee

P.J. Devereaux (Chair), Shrikant Bangdiwala, Flavia Borges, David Conen, Ingrid Copland, Gordon Guyatt, Maura Marcucci, Daniel Sessler, Jessica Vincent

## International Operations Committee

P.J. Devereaux (Chair), Shrikant Bangdiwala, Bruce Biccard, Flavia Borges, Matthew Chan, David Conen, Ingrid Copland, John Eikelboom, Gordon Guyatt, Kate Leslie, Vladimir Lomivorotov, Christian Meyhoff, Maura Marcucci, Thomas Painter, Joel Parlow, Toby Richards, Daniel Sessler, Wojciech Szczeklik, Vikas Tandon, Jessica Vincent, Denis Xavier

## Steering Committee

P.J. Devereaux (Chair), Pascal Alfonsi, Mohammed Amir, Shrikant Bangdiwala, Bruce Biccard, Flavia Borges, Matthew Chan, David Conen, Ingrid Copland, Emmanuelle Duceppe, John Eikelboom, Edith Fleischmann, Peter Gross, Gordon Guyatt, Raja Jayaram, Ydo Kleinlegtenbelt, Andrea Kurz, Giovanni Landoni, Kate Leslie, Vladimir Lomivorotov, Maria José Martinez Zapata, Yannick Lemanach, Michael McGillion, Christian Meyhoff, Maura Marcucci, Thomas Painter, Joel Parlow, Carisi Polanczyk, Toby Richards, Pavel Roshanov, Denis Schmartz, Daniel Sessler, Tim Short, Sadeesh Srinathan, Wojciech Szczeklik, Vikas Tandon, David Torres, Jessica Vincent, Chew Yin Wang, Dale Williams, Maria Wittmann, Denis Xavier, Salim Yusuf

## Event Adjudication Committee

David Conen (Chair), Teresa Cafaro, Nuria Ruiz López, Sandra Ofori, Michael Prystajecky, Andrés Valenzuela Suárez, Javiera Paz Vasquez, Michael Ke Wang, Ania Wludarczyk

## Data and Safety Monitoring Board (DSMB)

Ian Roberts (Chair), Dean Fergusson, Pierre Foex, Finlay McAlister

## Project Office Staff

Ingrid Copland, Sara Di Diodato, Rosemary Howe, Hannah MacDonald, Louise Mastrangelo, Natalie Mercuri, Sarah Molnar, Shirley Pettit, David Stillo, Trisha Southall, Jennifer Swanson, Makayla Tosh, Jessica Vincent

## Study Statisticians and Programmers

Kumar Balasubramanian, Shrikant I. Bangdiwala, Eric Deng, Tamara Marsden, Mark Molec, Olga Shestakovska, Robin Zhao

## National Leaders

Pascal Alfonsi (France), Mohammed Amir (Pakistan), Bruce Biccard (South Africa), Matthew Chan (Hong Kong), P.J. Devereaux (Canada), Edith Fleischmann (Austria), Andreas Hoeft (Germany), Raja Jayaram (United Kingdom), Ydo Kleinlegtenbelt (Netherlands), Giovanni Landoni (Italy), Vladimir Lomivorotov (Russia), Maria José Martinez Zapata (Spain), Christian Meyhoff (Denmark), Thomas Painter (Australia and New Zealand), Carisi Polanczyk (Brazil), Denis Schmartz (Belgium), Daniel Sessler (United States), Wojciech Szczeklik (Poland), David Torres (Chile), Chew Yin Wang (Malaysia), Maria Wittmann (Germany), Chaoran Wu (China), Denis Xavier (India)

## Participating Centres

**AUSTRALIA (557)** **–** *Royal Adelaide Hospital (202*): Louise de Prinse, Sam Lumb, Simon Macklin, Christine S. Osborn, Johanna Somfleth, Carolyn M. Wood; *Royal Melbourne Hospital (3*): Ned WR. Douglas; *Fiona Stanley Hospital (44*): Surbhi Malhotra, Lucy Glazov, Duncan Wright; *Royal Hobart Hospital (28*): Michael J. Challis, David R. Alcock, Robyn A. Seale; *Austin Health (14*): Philip J. Peyton, Sarah Baulch; *Dandenong Hospital, Monash Health (16*): Richard Bulach, Tina Maggio; *Princess Alexandra Hospital Southside Clinical Unit, University of Queensland (88*): David Thomas Highton, Stefan Saric, Adeel Aftab, Susanna Van Haeringen; *Flinders Medical Centre (7*): Jason P. Koerber; *Box Hill Hospital (17*): Libia E. Machado Munoz, Andrea Barton; *Prince of Wales Hospital (35*): Michael Bennett, Priscilla Marneros, Steve Cai; *John Hunter Hospital (30*): Ross K. Kerridge, Paul Healey, Jeanene M. Douglas; *Royal Perth Hospital (11*): Tomas B. Corcoran, Andrew J. Toner; *Westmead Hospital (1*): Alfred Tanaka Mahumani; *Goulburn Valley Health (4*): Nigel JW. Dunk; *Western Health Melbourne (23):* David Edmund Piers Bramley; Miriam Towns; *Peter MacCallum Cancer Centre and The University of Melbourne (13*): Bernhard Riedel, Kimberley Coleman; *Queen Elizabeth II Jubilee Hospital (21*): Sarah R. Bowman, Amos Moody.

**AUSTRIA (84)** **–** *Medical University of Vienna, 1090 Vienna, Austria (84*): Barbara Kabon; Christian Reiterer; Alexander Taschner; Nikolas Adamowitsch.

**BELGIUM (151)** **–** *CHU Brugmann (149*): Javad Bidgoli Seyed, Christiane Dzechi, Céline Van Lerberghe, Hélène Tatiana Besse, Ida Stany; *Cliniques Universitaires Saint Luc; Université Catholique de Louvain (2*): Mona Momeni.

**BRAZIL (71)** **–** *Hospital Santa Lúcia - Hospital do Coração de Poços de Caldas (52*): Ricardo R. Bergo, Frederico C. Dall’Orto, Gislayne R. Ribeiro, Keyla C. Bitencourt; *Hospital de Clínicas de Porto Alegre (19*): Luciana Cadore Stefani, Carisi Anne Polanczyk.

**CANADA (1872) –** *Hamilton General Hospital (318*): Susan O’Leary, John Harlock, Mohamed Panju, Kajenny Srivaratharajan, Betty Chui, Ryan Proc, Lisa Trombetta; *St. Joseph’s Healthcare (264*): Harsha Shanthanna, John Neary, Katie Connolly, Muammar Abdulrahman, Spencer Wikkerink, Faraaz Quraishi; *Juravinski Hospital (416*): Kelly Lawrence, Philip Joseph, Patrick Magloire, Amna Ahmed, Conor Cox, Krysten Gregus, Baha Alazzoni, Jacquie Hare; *Centre Hospitalier de l’Université de Montréal (257*): Nikola Joly, Francois-Martin Carrier, Luc Massicotte, Maxime Thibault, Brigitte Migneault, Francois Plante; *Kingston Health Sciences Centre (254*): Deborah A. Dumerton, Jason Erb, Jessica Shelley, Elorm Vowotor, Maria Karizhenskai, Emile Peponoulas; *University of Manitoba (47*): Biniam Kidane, Duane Funk; *University of Alberta Hospital (108*): Michael Jacka, Michelle Graham, James Greene, Derek Dillane, David Bigam; *Centre hospitalier Universitaire de Sherbrooke (4*): Marco Lefebvre; *Toronto General Hospital, University Health Network (26*): Stuart A. McCluskey, Ayach Nour, Jo Carroll; *University of Saskatchewan (21*): Michael R. Prystajecky, Peter D. Hedlin; *University of Calgary (102*): Kelly B. Zarnke, Linet Kiplagat, Shannon M. Ruzycki, Evan Minty, Prism S. Schneider; *University of Western Ontario (55*): Marko Mrkobrada, George K. Dresser, Jaclyn A. Ernst, George Nicolaou.

**CHILE (107) –** *Clínica Santa Maria (75*): Maite Fuentes, JavieraVasquez, Valentina Paz, Alejandra Cares; *Universidad de La Frontera (32*): Waldo Merino, Ruben Carrasco-Moyano, Claudio Carcamo.

**CHINA (52) –** *Shenzhen People’s Hospital, The First Affiliated Hospital of Southern University of Science and Technology (9*): Chaoran Wu; *The Fourth Affiliated Hospital, Harbin Medical University (37*): Sihua Qi, Yuying Xie, Mengdi Zhang; *Second Hospital of Anhui Medical University (5*): Ye Zhang.

**DENMARK (130) –** *Bispebjerg and Frederiksberg Hospital, University of Copenhagen (82*): Ossian N. Gundel, Marlene Søgaard, Diana B. Jensen, Viktor Moseholm; *Zealand University Hospital (32*): Ismail Gögenur, Nessn Azawi, Naomi Nadler; *Rigshospitalet, Copenhagen University (16*): Eske Kvanner Aasvang, Niels Højlund-Hansen.

**FRANCE (6) –** *GH Paris Saint Joseph* *(6*): Julia Lavergne.

**GERMANY (133) –** *University Hospital Bonn (21*): Mark Coburn, Claudia Neumann; *University Hospital, Düsseldorf (47*): Giovanna A.L. Lurati Buse, Sebastian Roth, Alexandra Stroda; *Medical Faculty University Hospital RWTH Aachen, Aachen, Germany (36*): Ana Kowark, Sebastian Ziemann, Linda Grüßer; *Klinik für Anästhesiologie, operative Intensivmedizin und postoperative Schmerztherapie (29*): Richard K. Ellerkmann, Holger Pohl, Anne Houben.

**HONGKONG (364) –** *The Chinese University of Hong Kong (364*): Gavin M. Joynt, Gordon YS. Choi, Wai Tat Wong, James YW. Lau, Beaker Fung, Eva Lee, Ka Yan Hui.

**INDIA (621) –** *Christian Medical College Hospital, Ludhiana (6*): Navneet Kumar Chaudhry; *Rahate Surgical Hospital (81*): Prashant Vitthalrao Rahate; *Sidhu Hospital, Doraha* *(77*): Ravinder Singh Sidhu, Gursaran Kaur Sidhu, Naveen Laiker, Abhitej Sidhu; *Sids Hospital & Research Center (170*): Dhaval Mangukiya, Jignesh Patel, Daxa Kachhadiya, Sanjay Thumar, Viral Parekh; *Sanjay Gandhi Post Graduate Institute Of Medical Sciences (27*): Sanjay Dhiraaj, Surendra Kumar Agarwal, Puneet Goyal; *Nanjappa Hospital (76*): Vishwanath B., Sachin.D.N.B, Veena Gopal, Shivaraj. D.M.; *Sengupta Hospital & Research Institute (43*): Shantanu P. Sengupta, Rajasi S. Sengupta, Suchitra S. Uikey; *Sumandeep Vidyapeeth an Institution Deemed to be University & Dhiraj Hopsital (7*): Vipul Gurjar; *ACE Hospital and Research Center (41*): Suresh B. Patankar, Suparn K. Khaladkar, Gururaj A. Padsalgi; *Bangalore Baptist Hospital* (70): K. Varghese Zachariah, Anil Kumar, Venkat Narasimhan N S.; *Government Td Medical college (7*): Alen Sigamani; *Government Medical College, Trivandrum (13*): Prem Haridas Menon, Rajeev Damodaran Nair Sarojini Amma; *Rabindranath Tagore International Institute of Cardiac Sciences (3*): Rahul Guhabiswas.

**ITALY (251) –** *IRCCS San Raffaele Scientific Institute (115*): Stefano Turi, Nora Di Tomasso, Giuseppe Dalessandro, Cristina Nakhnoukh, Carola Galbiati; *Galliera Hospital (72*): Claudia Brusasco, Giulia Orefice, Francesco Corradi, Michela Gandini; *IRCCS Galeazzi Orthopedic Institute (13*): Giuseppe De Blasio, Luigi Zagra; *IRCCS Orthopedic Institute Galeazzi (51*): Giorgio Oriani, Tancredi Guerrerio, Cesare Donarini, Marisa Iacurti.

**MALYSIA (654) –** *University of Malaya (302*): Tyng Yan Ng, Nur Azreen Hussain, Ri Qi Yeow, Yang Qun Choy, Xue Lin Chan, Nor Fadhilah Shahril, Farah Nadia Razali; *Port Dickson Hospital (52*): Hema Malini Manogharan, Siti Sufia Mohd Saleh, Teh Wahida Tusirin, Boon Tiang Lau; *Hospital Pulau Pinang (67*): Meng-Li Lee, Eric BK. Tang, Halimatun Muslim, Zettie Shuib; *Sarawak General Hospital (115*): Shu Ching Teo, Soon Keong Khaw, Yong Peng Lim, Samuel EH Tsan, Tze Ling Ng; *Sungai Buloh Hospital (11*): Chun Fai Chiang, Joyce Tien; *Hospital Kuala Lumpur (107*): Seleen Cheah, Su Yin Loo, Yeh Han Poh, Yoke Yin Woo, Siti Hawa Tahir.

**NETHERLANDS (165) –** *Deventer Hospital (151*): Ellie B.M. Landman, Stefan J.G Gelderman, Jan-Hein M.G. Cobben, Bas Staffhorst, Roel Mes; *Erasmus University Medical Center (14*): Felix van Lier, René van Bruchem.

**NEW ZEALAND (164) –** *Auckland City Hospital (67*): Elizabeth Maxwell, Douglas Campbell, Davina McAllister, Vikrant Singh; *Waikato Hospital (38*): Jonathan Termaat, Gay Mans, Kate Goldstone; *Counties Manukau District Health Board (59*): Jane Denman, Nicholas Lightfoot, Sue Olliff, Helen Houston.

**PAKISTAN (127) –** *Shifa Internatiopnal Hospital (91*): Tehreem Zahid, Sibgha Aimon, Sumeyya Azeem, Muhammad Uneeb; *Aga Khan University (36*): Mohsin Nazir, Rizwan Haroon Rashid, Khalid Ahsan.

**PORLAND (143) –** *Szpital Zakonu Bonifratrów w Krakowie (112*): Dorota Studzińska, Anna Włudarczyk, Marcin Jelinek, Gabriela Domaszewicz, Weronika Wajda; *Specjalistyczny szpital im E.Szczeklika w Tarnowie (15*): Pawel Grudzien, Anna Mirek; *University Hospital Zielona Góra (16*): Bartosz Kudliński, Jarosław Zawisza.

**RUSSIA (1840) –***E. Meshalkin National Medical Research Center (254*): Andrey A. Karpenko, Samandar M. Ismoilov, Olga A. Abubakirova, Galina A. Zeidlits, Mikhail N. Deryagin, Pavel E. Vedernikov; *Moscow Regional Clinical and Research Institute (149*): Alexey Muradovich Ovesov, Levan Bondaevich Berikashvili, Oleg Nikolaevich Gerasimenko, Nadezhda Vyacheslavovna Ermokhina, Ekaterina Vladimirovna Ryabova; *Northern State Medical University/City Hospital #1 of Arkhangelsk (207*): Vsevolod V. Kuzkov, Ayyaz Hussain, Alexey A. Smetkin, Darja V. Carionova, Yana Y. Ilyina, Dmitrii A. Volkov; *Tyumen State Medical University (36*): Tsiryateva S. B; *Novosibirsk State University (318*): Sergey V. Astrakov, Elena L. Neporada; *Saint Petersburg State University Hospital (811*): Alexey Y. Kulikov, Mikhail A. Alexeev, Konstantin S. Trukhin, Andrey A. Cherednichenko, Irina A. Andrianova, Svetlana S. Mikhailova, Andrey A. Sorokin, Elizaveta A. Leonova, Timur A. Dzhumatov ; *V. Negovsky Reanimatology Research Institute, Moscow Clinical Scientific Center n.a. A.S.Loginov (65*): Valerii Viacheslavovich Subbotin, Artem Nikolaevich Kusovlev, Kristina Kirilovna Kadanceva, Andrei Andreevich Klimov.

**SOUTH AFRICA (24) –** *Groote Schuur Hospital (21*): Ettienne Coetzee, Kobus Bergh; S*teve Biko Academic Hospital (1*): Sophie Mathijs; *Sefako Makgatho Health Sciences University (2*): Jan HR Becker.

**SPAIN (740) –** *Hospital Clinic Barcelona (53*): Oscar Comino-Trinidad, Carla Solé, Marina Vendrell, Beatriz Tena; *Hospital Universitari Vall d’Hebron (158*): Miriam de Nadal, Esther Cano, Maddalena Pasini, Patricia Galan, Alejandro Madrid; *Hospital de la Sta Creu i Sant Pau (192*): Marta Argilaga, Victoria Baños, Mireia Rodriguez, Inma India, Ana Maria Gomez-Caro; *Hospital Universitario Fundación Alcorcón (57*): JI García Sánchez, Rodrigo Molina, Diana Zamudio, Viviana Varón; *Hospital Universitari De Girona Dr J Trueta (4*): Marc Vives; *Ramón y Cajal University Hospital (113*): Ana Belén Serrano, Maria Gómez-Rojo, Ángel Candela-Toha; *Hospital Clínico Universitario de Valladolid (163*): Juan Berrocal-Cuadrado, Elvira Mateos-Álvarez, Adela Pereda-Manso, M. Carmen Cervera-Díaz, Nuria Ruiz-López.

**UNITED KINGDOM (76) –** *Chelsea & Westminster NHS Foundation Trust (9*): Marcela P. Vizcaychipi; *Medway NHS Trust U.K (65*): Tarannum Rampal, Arystarch Miron Makowski, Iram Ahmed, Thyra Kyere-Diabour; *West Middlesex University Hospital (Chelsea and Westminister Foundation Trust) (2*): Surendini Thayaparan.

**USA (1041) –** *MD Anderson Cancer Center (127*): Juan P. Cata, German Corrales, Ronaldo Purugganan, Peter Norman, Olakunle Idowu; *Cleveland Clinic (213*): Azfar K. Niazi, Andrew Volio, Daniel Kim, Paul Minko, Dilara S. Khoshknabi, David Hoying; *Cleveland Clinic (262*): Mauro Bravo, Jorge Andres Araujo Duran, Hani Essber, Ruben Agudelo Jimenez, Lauretta Mosteller, Alexis Skolari; *Cleveland Clinic (13*): Partha J Saha, Kavita Elliott; *Columbia University Irving Medical Center (2*): Gebhard Wagener; *Wake Forest School of Medicine (48*): Scott Alan Miller, Bruce Cusson, Lynne Harris; *University of North Carolina, Chapel Hill (129*): Priya A. Kumar, Harendra Arora, Meena Bhatia; *The Ohio State Unversity Wexner Medical Center (110*): Tristan E. Weaver, Sergio D. Bergese, Alberto A. Uribe, Joshua D. Pasek, Jaume Borell-Vega; *Cleveland Clinic Florida (103)* Steven C. Minear, Camila Teixeira, Syed Sohaib Nasim, Tatiana Jamroz, Santiago Luis; *Oregon Health & Science University (19*): Sydney E. Rose, Vincent Pinkert; *Rhode Island Hospital, The Warren Alpert Medical School of Brown University (5*): Denis V. Snegovskikh; *Yale School of Medicine* *(10*): Shamsuddin Akhtar.
